# Heparin-binding epidermal growth factor and fibroblast growth factor 2 rescue Müller glia-derived progenitor cell formation in microglia- and macrophage-ablated chick retinas

**DOI:** 10.1242/dev.202070

**Published:** 2023-12-06

**Authors:** Heithem M. El-Hodiri, James R. Bentley, Alana G. Reske, Olivia B. Taylor, Isabella Palazzo, Warren A. Campbell, Nicklaus R. Halloy, Andy J. Fischer

**Affiliations:** ^1^ Department of Neuroscience, College of Medicine, The Ohio State University, Columbus, OH 43221, USA; ^2^ Solomon Snyder Department of Neuroscience, Johns Hopkins University, Baltimore, MD 21205, USA

**Keywords:** Müller glia, Cell signaling, Microglia, Retinal regeneration, scRNA-seq

## Abstract

Recent studies have demonstrated the impact of pro-inflammatory signaling and reactive microglia/macrophages on the formation of Müller glial-derived progenitor cells (MGPCs) in the retina. In chick retina, ablation of microglia/macrophages prevents the formation of MGPCs. Analyses of single-cell RNA-sequencing chick retinal libraries revealed that quiescent and activated microglia/macrophages have a significant impact upon the transcriptomic profile of Müller glia (MG). In damaged monocyte-depleted retinas, MG fail to upregulate genes related to different cell signaling pathways, including those related to Wnt, heparin-binding epidermal growth factor (HBEGF), fibroblast growth factor (FGF) and retinoic acid receptors. Inhibition of GSK3β, to simulate Wnt signaling, failed to rescue the deficit in MGPC formation, whereas application of HBEGF or FGF2 completely rescued the formation of MGPCs in monocyte-depleted retinas. Inhibition of Smad3 or activation of retinoic acid receptors partially rescued the formation of MGPCs in monocyte-depleted retinas. We conclude that signals produced by reactive microglia/macrophages in damaged retinas stimulate MG to upregulate cell signaling through HBEGF, FGF and retinoic acid, and downregulate signaling through TGFβ/Smad3 to promote the reprogramming of MG into proliferating MGPCs.

## Teaser

**Summary:** Signals provided by reactive microglia and macrophages promote the formation of Muller glia-derived progenitors by activated HBEGF and FGF pathways.

## INTRODUCTION

The process of retinal regeneration varies between vertebrate species. In fish retinas, neuronal regeneration is a robust process that restores visual function following injury, whereas this process is far less robust in birds and absent in mammals (Hitchcock and Raymond, 1992; Karl et al., 2008; Raymond, 1991). Müller glia (MG) have been identified as the cell of origin for progenitors in regenerating retinas (Bernardos et al., 2007; Fausett and Goldman, 2006; Fausett et al., 2008; Fischer and Reh, 2001; Ooto et al., 2004). In the retina, MG are the most prevalent type of support cell and these cells normally provide metabolic support, structural support, synaptic support and contribute to recycling of visual pigments (Reichenbach and Bringmann, 2013). Neuronal damage, certain growth factors or drug treatments can stimulate MG to become activated, de-differentiate, upregulate genes associated with progenitor cells, proliferate and produce new neurons (Fischer and Bongini, 2010; Gallina et al., 2014; Wan and Goldman, 2016).

In mammalian retinas, the reprogramming of MG into progenitor-like cells requires significant stimulation, such as forced expression of *Ascl1*, inhibition of histone deacetylases, and neuronal damage, to produce a few functional neurons (Jorstad et al., 2017; Pollak et al., 2013; Ueki et al., 2015). Alternatively, inducible, targeted deletion of *Nfia*, *Nfib* and *Nfix* in mature MG, combined with intra-ocular injections of insulin+FGF2 and neuronal damage, stimulates the reprogramming of MG into progenitors that produce cells resembling inner retinal neurons (Hoang et al., 2020). In damaged chick retinas, MG form numerous proliferating MG-derived progenitor cells (MGPCs), but the neurogenic capacity of MGPCs is very limited with relatively few progeny differentiating into neurons (Fischer and Reh, 2001, 2003). Identification of the mechanisms that activate or suppress the formation of MGPCs and suppress the neurogenic potential of MGPCs is required to harness the regenerative potential of MG in warm-blooded vertebrates.

The responses of immune cells, namely microglia and macrophages, to damage have a profound impact upon the ability of MG to reprogram into progenitor-like cells. For simplicity, hereafter microglia and macrophages will be collectively referred to as microglia because these cell types are not easily distinguished within the retina. In zebrafish, microglia influence the ability of MG to regenerate retinal neurons; the absence of reactive microglia slows the process of neuronal regeneration (Huang et al., 2012; White et al., 2017). In mouse, ablation of microglia enhances the generation of neuron-like cells from Ascl1-overexpressing MG in the retina (Todd et al., 2020). Reactive microglia rapidly upregulate pro-inflammatory cytokines to damaged retinas (Todd et al., 2019), and these cytokines activate NFκB signaling in MG, which, in turn, suppresses the neurogenic potential of Ascl1-overexpressing MG (Palazzo et al., 2022). In chick, ablation of microglia from the retina blocks the formation of proliferating MGPCs in damaged retinas (Fischer et al., 2014). Further, we previously found that the impact of NFκB signaling on the formation of MGPCs is reversed in the absence of reactive microglia; activation of NFκB suppresses MGPC formation, whereas in the absence of microglia activation of NFκB starts the process (Palazzo et al., 2020). Accordingly, the purpose of this study was to investigate the mechanisms underlying the failure of MGPC formation in chick retinas in which the microglia/macrophages have been ablated.

## RESULTS

### Single-cell RNA-sequencing analyses of MG from retinas with and without microglia

For simplicity, hereafter, we refer to retinal microglia and infiltrating macrophages as ‘microglia’ because the CD45 antibodies used to identify these cells do not distinguish between microglia and macrophages. Retinal microglia were depleted by a single intravitreal injection of clodronate liposomes, which effectively destroys >95% of microglia within 3 days of treatment. Fluorescently labeled clodronate liposomes accumulate only at the vitread surface of the retina and are taken up only by microglia, whereas the MG appear unaffected (Fischer et al., 2014; Zelinka et al., 2012). We also tested CSF1R antagonists, PLX5622 and BLZ945, in chick with the intent of ablating microglia in the retina. However, although chick microglia highly express CSF1R, these antagonists did not deplete retinal microglia (Fig. S4).

**Fig. 1.**
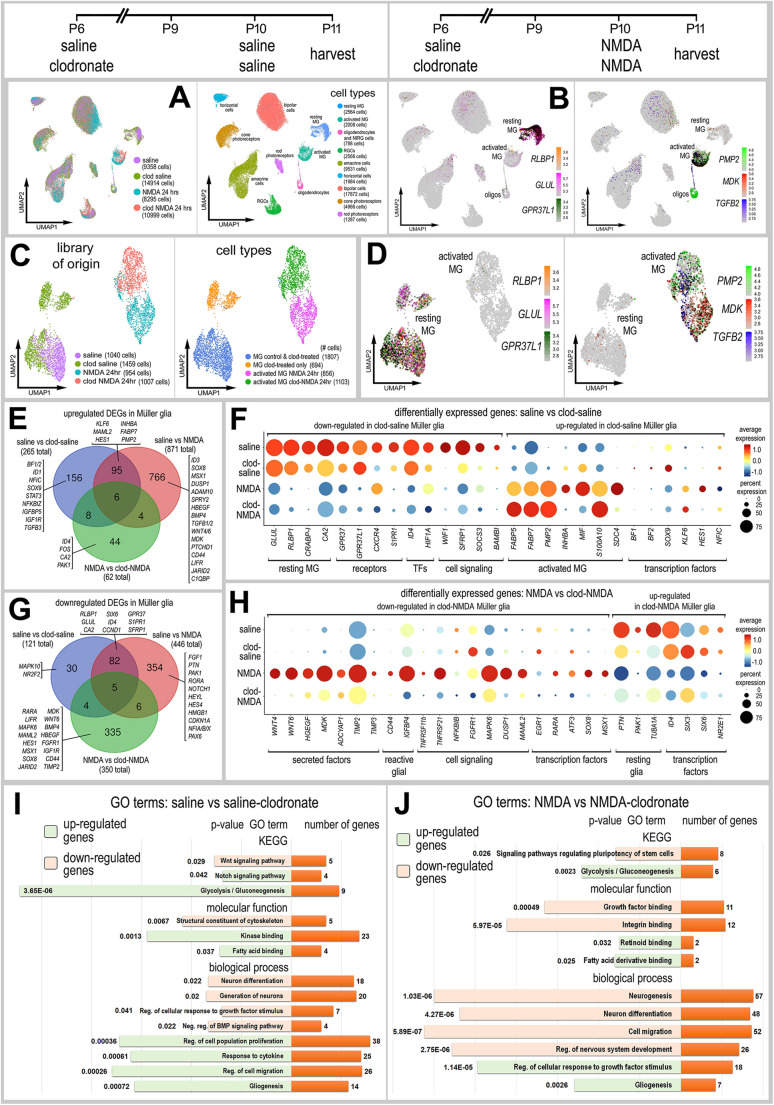
**scRNA-seq of normal and damaged retinas with and without microglia.** Retinas were treated with saline or clodronate liposomes at P6, followed by saline or NMDA at P10, and tissues harvested at 24 h after the last injection. (A) UMAP ordering of cells for libraries of origin and distinct clusters. (B,D) In UMAP heatmap plots, resting MG were identified by elevated expression of *RLBP1*, *GLUL* and *GPR37L1*, and activated MG were identified by elevated expression of *PMP2*, *MDK* and *TGFB2*. (C,D) MG were bioinformatically isolated and re-embedded in UMAP plots. (E,G) Lists of DEGs were generated (Tables S1-S3) for MG from retinas treated with saline versus clodronate+saline, saline versus NMDA, and NMDA versus clodronate+NMDA. Numbers of up- and downregulated DEGs are plotted in Venn diagrams with representative unique genes listed. (F,H) Dot plots illustrating the percentage of expressing MG (size) and significant (*P*<0.01) changes in expression levels (heatmap) for genes in MG from retinas treated with saline versus saline+clodronate (F) and NMDA versus NMDA+clodronate (H). (I,J) GO enrichment analysis was performed for lists of DEGs in MG treated with saline±clodronate and NMDA±clodronate. Gene modules for upregulated (green) and downregulated (peach) genes were grouped by GO category with *P*-values and numbers of genes in each category. Clod, clodronate; KEGG, Kyoto Encyclopedia of Genes and Genomes; Neg., negative; oligos, oligodendrocytes; Reg., regulation; TF, transcription factors.

We generated four single-cell RNA-sequencing (scRNA-seq) libraries: (1) control undamaged retina, (2) undamaged retinas with microglia depleted, (3) NMDA-damaged retinas at 24 h after treatment, and (4) NMDA-damaged retinas at 24 h after treatment with microglia depleted. Although we have previously extracted discrete information from these scRNA-seq libraries regarding the impact of microglia on the expression of NFκB-signaling components in MG (Palazzo et al., 2020), herein we provide the first in-depth analyses of these datasets. Uniform manifold approximation and projection (UMAP) plots of aggregated libraries (43,566 total cells) revealed distinct clusters of cell types, with neuronal types clustered together regardless of treatment and MG forming distinct clusters that correlated with treatments (Fig. 1A,B). Clusters of retinal cells were identified based on cell-distinguishing markers, as described in Materials and Methods. Resting MG were identified based on patterns of expression for *GLUL*, *RLBP1* and *GPR37L1*, and activated MG were identified based on expression of *PMP2*, *TGFB2* and *MDK* (Fig. 1B-D). Very few (<50) microglia were captured from control retinas, and these cells were filtered based on low genes/unique molecular identifiers (UMIs) per cell; this may be because the libraries were generated using Chromium 3′ V2 reagents, which have limited sensitivity compared with more recently developed reagents. We bioinformatically isolated MG and re-ordered these cells in a UMAP plot. Approximately 1000 MG from each treatment group formed distinct clusters of cells that were segregated into resting and activated MG (Fig. 1C,D). We generated lists of differentially expressed genes (DEGs) that were up- or downregulated in MG in undamaged and damaged retinas when the microglia were ablated (Fig. 1E,G; Tables S1-S3). The depletion of microglia in undamaged retinas caused MG to downregulate many (121) genes associated with resting MG, including different receptors, transcription factors, and components of different cell signaling pathways (Fig. 1F,G). Many (87) of these genes were also downregulated by MG in NMDA-damaged retinas (Fig. 1G). The depletion of microglia in undamaged retinas caused MG to significantly upregulate many (265) genes associated with activated MG, including secreted factors, pro-inflammatory cell signaling components, genes involved in fatty acid metabolism and transcription factors known to promote a glial phenotype (Fig. 1E,F).

There is some evidence that MG in the fish retina can adopt phagocytic functions when microglia are ablated (Thiel et al., 2022). Accordingly, we probed scRNA-seq libraries for genes associated with the phagocytic cup and early endosomes in MG in damaged retinas with and without microglia. MG did not upregulate any receptors found in phagocytic cups or genes associated with early endosomes in damaged retinas in which microglia had been ablated. These genes were either not expressed (or expressed at very low levels) or were unaffected by the ablation of microglia, with the exception of *BIN1* and *RABEP1* (only two out of 15 genes; *AP4M1* was not significantly different) (Fig. S1).

We identified many DEGs in MG in damaged retinas that were affected by the absence of microglia (Fig. 1E,G,H). Relatively few (62) upregulated DEGs were identified, whereas numerous (350) downregulated DEGs were identified in MG in damaged retinas missing microglia (Fig. 1G). Downregulated DEGs included many secreted factors known to stimulate the proliferation of MGPCs, markers of reactive glia, and genes associated with NFκB signaling (*TNFRSF11B*, *TNFRSF21*, *NFKBIB*), MAPK signaling (*FGFR1*, *MAPK4*, *DUSP1*) and Notch signaling (*MAML2*) (Fig. 1G,H). Among the most significantly downregulated genes in MG in damaged retinas missing microglia encoded secreted factors known to stimulate the formation of proliferating MGPCs, including Wnt ligands (Gallina et al., 2015), HBEGF (Todd et al., 2015) and midkine (MDK) (Campbell et al., 2021a).

We performed Gene Ontology (GO) enrichment analyses of lists of up- and downregulated genes in MG in undamaged retinas without microglia. Depletion of microglia from undamaged retinas stimulated MG to downregulate gene modules associated with Wnt signaling, BMP signaling and neurogenesis (Fig. 1I). By comparison, depletion of microglia from undamaged retinas stimulated MG to upregulate gene modules associated with Notch signaling, fatty acid binding, glycolysis, cellular proliferation/migration and gliogenesis (Fig. 1I). GO enrichment analyses of lists of up- and downregulated genes in MG in damaged retinas without microglia revealed upregulated gene modules associated with glycolysis, fatty acid binding and gliogenesis, similar to the gene modules identified in undamaged retinas missing microglia (Fig. 1J). By comparison, depletion of microglia from damaged retinas stimulated MG to downregulate gene modules associated with regulation of stem cell pluripotency, growth factor/integrin binding and neuronal development/differentiation (Fig. 1J).

### Implied ligand–receptor interactions that change when microglia are ablated

**Fig. 2.**
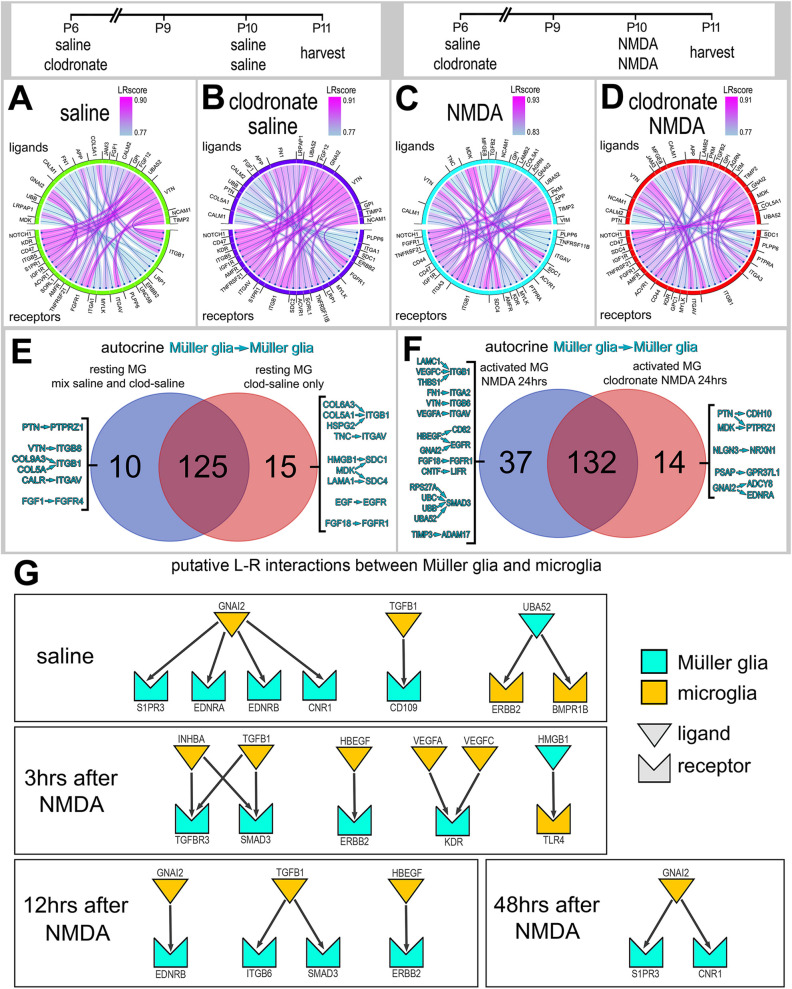
**Inferred autocrine ligand-receptor (LR) interactions.** (A-D) SingleCellSignalR was used to identify putative LR interactions. Chord diagrams illustrate the top 30 most significant autocrine LR interactions between MG in retinas treated with saline (A), clodronate+saline (B), NMDA (C) and clodronate+NMDA (D). (E,F) Autocrine interactions were compared for saline versus clodronate+saline and NMDA versus clodronate+NMDA. Venn diagrams illustrate the numbers of unique and common LR interactions between treatment groups, and list representative LR interactions unique to MG in undamaged retinas with and without microglia (E) and NMDA damaged retinas with and without microglia (F). (G) Representative LR interactions between microglia and MG for undamaged and damaged retinas at different times after NMDA treatment. We analyzed an aggregate library with neurons and glia from control undamaged retinas and retinas at 3, 12 and 48 h after NMDA, which included a total of 1134 microglia and 4700 MG for SingleCellSignalR analyses.

We next bioinformatically isolated the MG and re-embedded these cells to probe for putative ligand–receptor (LR) interactions using SingleCellSignalR (Cabello-Aguilar et al., 2020). We started by analyzing autocrine signaling among MG that might be affected by the ablation of microglia/macrophages. Resting MG included cells for saline and clodronate treatment groups; activated MG included cells from NMDA and clodronate+NMDA treatment groups. The numbers of LR interactions (significant upregulation of putative ligand and receptor) among MG in different treatment groups varied between 135 and 169 total interactions (Table S6). We performed analyses on MG from each treatment group and compared changes across the most significant LR interactions (Fig. 2A-D). Autocrine LR interactions unique to MG in undamaged retinas included signaling through different integrins, FGFR4 and pleotrophin (PTN)-PTPRZ1 (Fig. 2A,E). By comparison, LR interactions unique to MG in undamaged retinas missing microglia included signaling through different ligand–integrin pairs, MDK/SDC1/4, EGF/EGFR and FGF18/FGFR1 (Fig. 2B,E). LR interactions unique to MG in damaged retinas with microglia included HBEGF/EGFR, FGF18/FGFR1, and SMAD3 (Fig. 2C,F). By comparison, LR interactions unique to MG in damaged retinas missing microglia included signaling through PTN/MDK/PTPRZ1, the endothelin receptor EDNRA, and the orphan receptor GPR37L1, which is normally highly expressed by resting MG (Fig. 2D,F). Collectively, these findings indicate that microglia have a significant impact on potential autocrine signaling among MG in damaged and undamaged retinas.

To identify putative paracrine LR interactions between MG and microglia, we bioinformatically isolated MG and microglia from scRNA-seq libraries that we previously generated and described in detail (Campbell et al., 2021b, 2022, 2023). These libraries were generated from cells captured from normal and NMDA-damaged retinas at 3, 12 and 48 h after treatment (Fig. 2G). These libraries contained more than 1100 microglia and 4700 MG to permit analyses of putative paracrine LR interactions. We identified LR interactions between microglia and MG in undamaged retinas involving ligands (CNR1, TGFB1) and receptors (ERBB2, BMPR1B) (Fig. 2G; Fig. S2) known to influence the reprogramming of MG (Campbell et al., 2021b; Todd et al., 2015, 2017). In NMDA-damaged retinas, we identified LR interactions between microglia and MG involving TGFB1/TGFBR3/SMAD3 and HBEGF/ERBB2 (Fig. 2G; Fig. S1), pathways known to inhibit or activate, respectively, the formation of MGPCs (Todd et al., 2015, 2017). In addition, we found LR interactions involving VEGF ligands and the endothelin receptors EDNRA and EDRNB (Fig. 2G). A recent study in zebrafish retinas indicates that expression of Vegfa is induced in MG by reactive microglia/macrophages to promote the reprogramming of MG into proliferating MGPCs (Mitra et al., 2022). Collectively, the analysis of LR interactions between microglia and MG suggests a complex exchange of signals between these glial cells, which includes several pathways that have been implicated in the formation of MGPCs.

### Wnt signaling and the formation of MGPCs in damaged retinas missing microglia

**Fig. 3.**
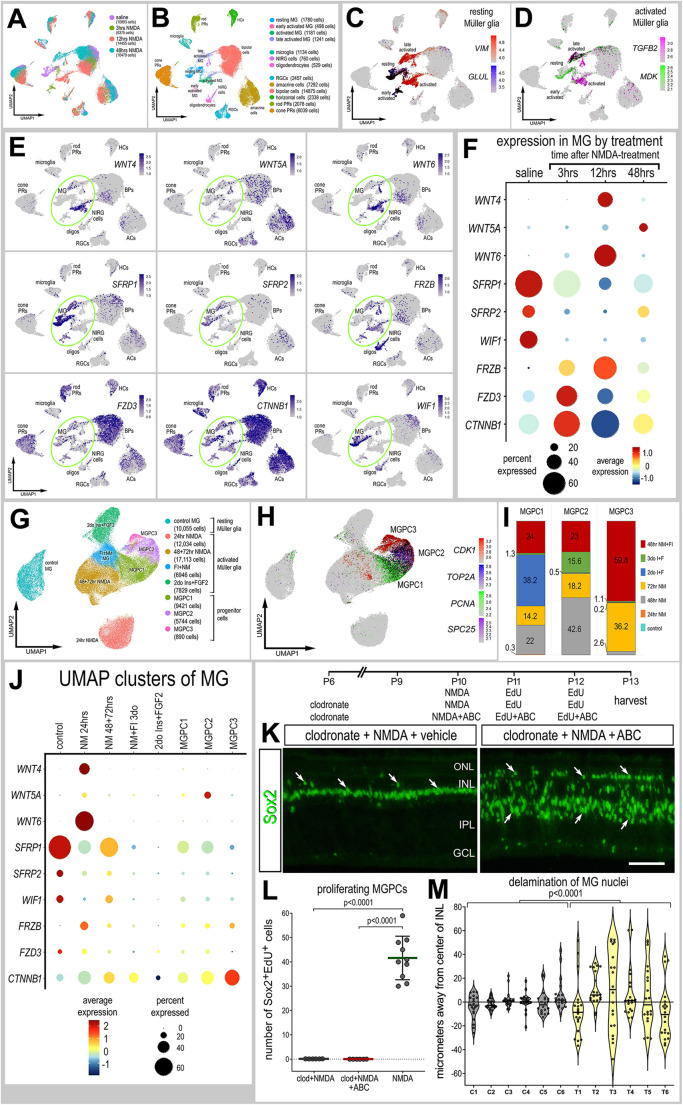
**Patterns of expression of Wnt-related genes.** (A) scRNA-seq was used to identify patterns and levels of expression of Wnt-related genes in control and NMDA-damaged retinas at 3, 12 and 48 h after treatment. (B) UMAP clusters of cells were identified based on well-established patterns of gene expression (see Materials and Methods). (C,D) MG were identified by expression of *VIM* and *GLUL* in resting MG (C), and *TGFB2* and *MDK* for activated MG (D). Each dot represents one cell and black dots indicate cells that express two or more genes. (E) UMAP heatmap plots illustrating patterns and levels of expression for Wnt-related genes. Green ovals highlight clusters of MG. (F,J) Dot plots illustrating average expression (heatmap) and percent expressed (dot size) in MG. (G-J) MG were bioinformatically isolated from scRNA-seq libraries of control retinas, retinas at 24, 48 and 72 h after NMDA treatment, retinas treated with two or three doses of insulin and FGF2, and retinas treated with insulin, FGF2 and NMDA at 48 h after NMDA for a total of 70,032 MG. (G,H) MG formed distinct UMAP clusters that correlated with different treatments (G), and MGPCs clustered by progression through the cell cycle (H). (I) Clusters of MGPCs were occupied by cells from different treatment groups. (K) We applied clodronate liposomes at P6, NMDA a cocktail of GSK3β inhibitors at P10, EdU±GSK3β inhibitors at P11 and P12, and harvested retinas at P13. Retinal sections were labeled for EdU accumulation and immunolabeled for Sox2 (green; K). Inhibition of GSK3β to stimulate Wnt signaling failed to stimulate MGPCs proliferation, but induced delamination of MG nuclei. Arrows indicate the nuclei of MG. Scale bar: 50 µm. (L) Mean MGPC proliferation (±s.d.); significance of difference (*P*-values) was determined using a Mann–Whitney *U*-test with Bonferroni correction. (M) Violin plot representing MG nuclear delamination; significance of difference (*P*-values) was determined using one-way ANOVA. In L,M, each dot represents one biological replicate. ABC, GSK3β inhibitor cocktail; C, control; GCL, ganglion cell layer; INL, inner nuclear layer; IPL, inner plexiform layer; ONL, outer nuclear layer; T, treated.

The Wnt ligands *WNT4* and *WNT6* are among the most significantly downregulated genes in MG in damaged retinas in which microglia were ablated (Fig. 1G,H; Table S2). Activation of Wnt signaling is known to stimulate the formation of proliferating MGPCs in the retinas of fish (Ramachandran et al., 2011), chicks (Gallina et al., 2015), and rodents (Osakada et al., 2007; Yao et al., 2016). Accordingly, we probed for expression levels of Wnt-related genes in scRNA-seq libraries of retinas obtained at 3, 12 and 48 h after NMDA treatment, as described for the LR interactions in Fig. 2G, or UMI. We analyzed a total of 42,202 cells, including 4700 MG (Fig. 3A,B). UMAP ordering of cells formed discrete clusters of neurons according to cell type and MG according to treatment (Fig. 3A,B). MG were identified based on expression of *VIM*, resting MG on expression of *GLUL* and activated MG were identified based on expression of *MDK* and *TGFB2* (Fig. 3C,D). *WNT4* and *WNT6* were expressed at low levels by resting MG and activated MG at 3 and 48 h after NMDA, but were highly expressed by activated MG at 12 h after NMDA (Fig. 3E,F). By comparison, *WNT5A* was upregulated by MG at 48 h, but was also expressed by some retinal ganglion, amacrine and bipolar cells (Fig. 3E,F). The Wnt inhibitors *SFRP1*, *SFRP2* and *WIF1* were highly expressed by resting MG and rapidly downregulated by 3 h after NMDA, remaining low at 48 h after treatment (Fig. 3E,F). By contrast, the Wnt inhibitor *FRZB* was low in resting MG and elevated at 3 and 12 h after NMDA, and was highly expressed by oligodendrocytes and non-astrocytic inner retinal glial (NIRG) cells (Fig. 3E,F). The Wnt receptor *FZD3* and transcriptional effector *CTNNB1* (β-catenin) were rapidly upregulated at 3 h, downregulated at 12 h, and back up at 48 h after NMDA treatment (Fig. 3E,F). Collectively, these findings suggest that Wnt-related genes are rapidly (3 h or less) and transiently up- or downregulated in MG following damage to retinal neurons.

To gain a more comprehensive understanding of patterns of expression of Wnt-related genes, we probed a large aggregate scRNA-seq library of MG (>70,000 cells) generated as described in previous studies (Campbell et al., 2021a,b; El-Hodiri et al., 2022; Hoang et al., 2020). We bioinformatically isolated the MG from retinas treated with saline, 24/48/72 h NMDA, two or three doses of insulin+FGF2, and 48 h NMDA+insulin+FGF2 (Fig. 3G,H), Resting MG and MG at 24 h after NMDA formed two distinct clusters of cells, whereas activated MG from different treatment groups and MGPCs formed a continuum of cells based, in part, on expression of cell cycle regulators (Fig. 3G-J). *WNT4* and *WNT6* were exclusively upregulated by the MG from 24 h after NMDA treatment (Fig. 3J). *WNT5A* was most highly expressed in the MGPC2 cluster, which was predominantly occupied by MG from retinal libraries from 48 and 72 h after NMDA, two doses of insulin+FGF2, and 48 h after NMDA+insulin+FGF2 (Fig. 3I,J). The Wnt inhibitors *SFRP1*, *SFRP2* and *WIF1* were highest in resting glia and reduced in activated MG and MGPCs from all treatments (Fig. 3J). *FRZB* was highest in MG at 24 h after NMDA and was decreased in all other MG (Fig. 3J). The Wnt receptor *FZD3* was relatively high in resting MG and decreased in all other treatment groups, except for the MG at 3 h after NMDA represented in the other aggregate library (Fig. 3F,J). *CTNNB1* (β-catenin) was most highly expressed in the MGPC3 cluster, which was predominantly occupied by MG from retinal libraries from 72 h after NMDA and 48 h after NMDA+insulin+FGF2 (Fig. 3I,J). Collectively, these findings suggest that Wnt-related genes are transiently up- or downregulated in MG following treatments that stimulate the formation of MGPCs, regardless of the presence of retinal damage.

We next tested whether intra-ocular injections of GSK3β inhibitors rescued the deficit of MGPC proliferation in damaged retinas missing reactive microglia. The biological activity of recombinant Wnt ligands remains uncertain because these proteins do not have the correct conformational folding that is necessary for biological function. Therefore, we focussed our efforts on stimulating Wnt signaling by targeting the transduction pathway (not shown). We have previously shown that nuclear localization of β-catenin is maximal in proliferating MGPCs at 2-3 days after NMDA treatment (Gallina et al., 2016). Accordingly, we injected a cocktail of GSK3β inhibitors (‘ABC’) that have been shown to stimulate the accumulation of nuclear β-catenin and the proliferation of MGPCs in damaged retinas (Gallina et al., 2016). We found that the cocktail of GSK3β inhibitors failed to stimulate the proliferation of MGPCs; these cells did not accumulate 5-ethynyl-2′-deoxyuridine (EdU) (Fig. 3L) or express phosphohistone H3 and neurofilament (Fig. S4C,D). However, application of GSK3β inhibitors did cause widespread delamination of MG nuclei (Fig. 3K-M). In all treated individuals, we observed a significant migration of Sox2-positive MG nuclei away from the middle of the inner nuclear layer (INL) (Fig. 3K,M). To better understand the effects of GSK3β inhibitors on MG, we investigated patterns of expression of the glial markers Pax2, Pax6 and glutamine synthetase (GS). Pax2 and Pax6 are expressed at relatively low levels in resting MG, with Pax2 prominently expressed by MG in central regions of the retina, and levels of expression are increased in response to neuronal damage (Stanke et al., 2010). By contrast, GS is expressed at high levels by resting MG and is downregulated in response to neuronal damage (Campbell et al., 2021a,b; Fischer and Reh, 2001; Fischer et al., 2002). We found that GSK3β inhibitors had no significant effect on levels of Pax2, whereas levels of GS were significantly decreased in MG, and levels of Pax6 appeared to be increased in nuclei of delaminated cells (Fig. S3). Collectively, these findings indicate that activated microglia in damaged retinas stimulate MG to highly upregulate Wnt ligands, and Wnt signaling may normally stimulate the migration and de-differentiation of MG in damaged retinas, but this is not sufficient to stimulate the proliferation of MGPCs unless microglia-dependent signals are provided.

### Exogenous FGF2 rescues the failure of MGPC proliferation in damaged retinas missing microglia

**Fig. 4.**
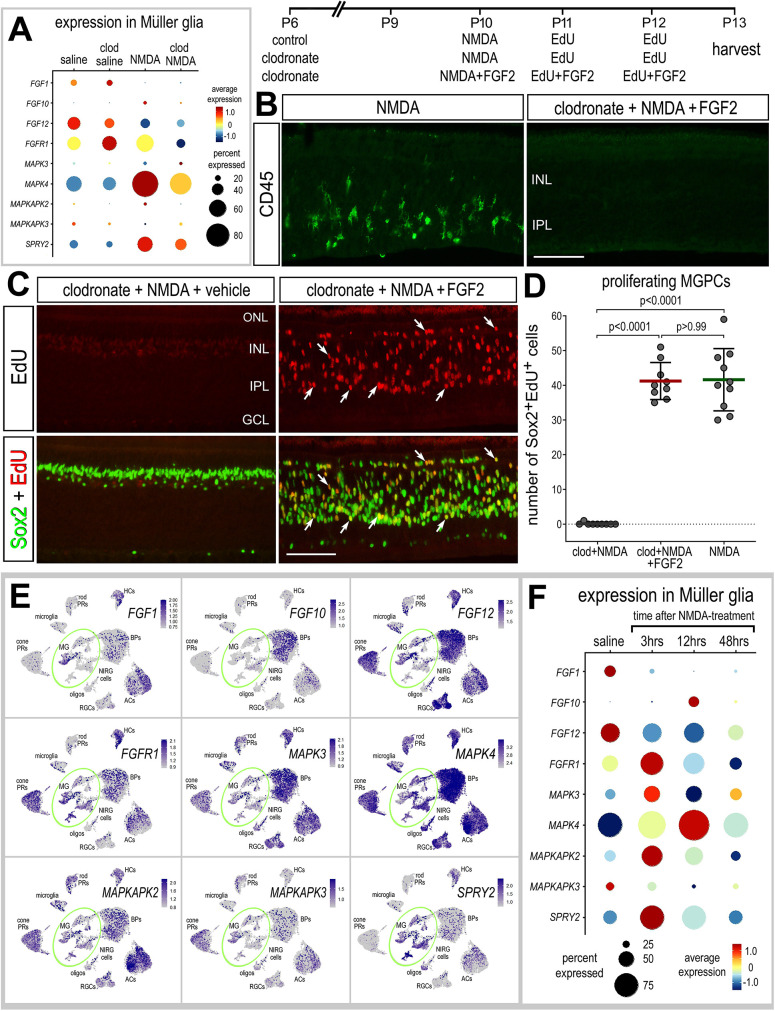
**Activation of FGF2 rescues the deficit in MGPC proliferation in damaged retinas missing microglia.** (A,E,F) Patterns and levels of expression of FGF/MAPK-related genes in scRNA-seq libraries of retinas treated with saline±clodronate and NMDA±clodronate (A), or different times after NMDA treatment (E,F). UMAP heatmap plots (E) and dotplot (F) illustrate patterns, percent expressed and levels of expression for FGF/MAPK-related genes in retinal neurons and glia in control retinas and retinas at 3, 12 and 48 h after NMDA treatment. The dot plots in A and F illustrate expression levels (heatmap) and percent expressed (dot size) for different genes in MG. (B-D) Retinal sections were labeled for EdU accumulation (red; C) and immunolabeled for Sox2 (green; C) and CD45 (green; B). Arrows indicate the nuclei of MG. The graph in D illustrates the mean (±s.d.) and each dot represents one biological replicate. Significance of difference (*P*-values) was determined using Mann–Whitney *U*-test with Bonferroni correction. Scale bars: 50 µm.

Analyses of scRNA-seq libraries of MG from undamaged and NMDA-damaged retinas with and without microglia indicated that many components of FGF/MAPK signaling were upregulated by MG in damaged retinas and this upregulation is diminished when microglia are absent (Fig. 4A; Tables S1, S5). These components included *FGFR1*, *MAPK4*, *MAPKAPK2* and *SPRY2* (Fig. 4A). By contrast, *MAPKAPK3* was significantly increased in MG in damaged retinas missing microglia (Fig. 4A; Tables S2, S5). By comparison, levels of *FGF1*, *FGF12* and *MAPKAPK3* were significantly decreased in NMDA-damaged retinas, but unaffected by the ablation of microglia (Fig. 4A; Tables S2, S5). MAPK-related factors in MG were not significantly affected by the ablation of microglia in undamaged retinas (Fig. 4A; Tables S1, S5).

A single intravitreal injection of clodronate liposomes effectively eliminated all microglia and any infiltrating macrophages from the retina for duration of experimentation (Fig. 4B). This is consistent with previous reports that clodronate liposomes selectively accumulate in reactive microglia near the vitread surface of the retina and deplete monocytes from the chick retina for at least 28 days (Fischer et al., 2014; Zelinka et al., 2012). We tested whether FGF2 rescued the deficit in MGPC proliferation in damaged retinas missing microglia. Injections of FGF2 with and following NMDA administration significantly increased numbers of proliferating MGPCs in retinas missing microglia (Fig. 4C,D). The number of proliferating MGPCs in clodronate/NMDA/FGF2-treated retinas was not significantly different from numbers seen in retinas treated with NMDA alone (Fig. 4D), suggesting a complete rescue. In addition, the proliferation of MGPCs was accompanied by widespread delamination of Sox2-positive nuclei in FGF2-treated damaged retinas missing microglia (Fig. 4C).

To investigate the changes in expression over time, we probed for expression levels of FGF/MAPK-related genes in the retina and MG in scRNA-seq libraries generated shortly (3 or 12 h) after NMDA treatment. *FGF1*, *FGF12*, *FGFR1*, *MAPK1* (*MAPK3*), *MAPK4*, *MAPKAPK2*, *MAPKAPK3* and *SPRY2* were widely expressed by retinal neurons and glia, whereas *FGF10* was predominantly expressed by bipolar cells and some amacrine cells (Fig. 4E). *FGF1*, *FGF12* and *MAPKAPK3* were rapidly (<3 h) downregulated by MG following NMDA treatment (Fig. 4E,F; Tables S4, S5). By contrast, *FGF10*, *FGFR1*, *MAPK1*, *MAPK4*, *MAPKAPK2* and *SPRY2* were rapidly (3-12 h) upregulated by MG following NMDA treatment (Fig. 4E,F; Tables S4, S5). These genes were downregulated by 12 or 48 h after NMDA treatment (Fig. 4E; Tables S4, S5). Collectively, these findings are consistent with the notion that MAPK signaling is rapidly activated in MG following neuronal damage and is crucially important to the reprogramming of MG into MGPCs.

### Exogenous HBEGF rescues the failure of MGPC proliferation in damaged retinas missing microglia

**Fig. 5.**
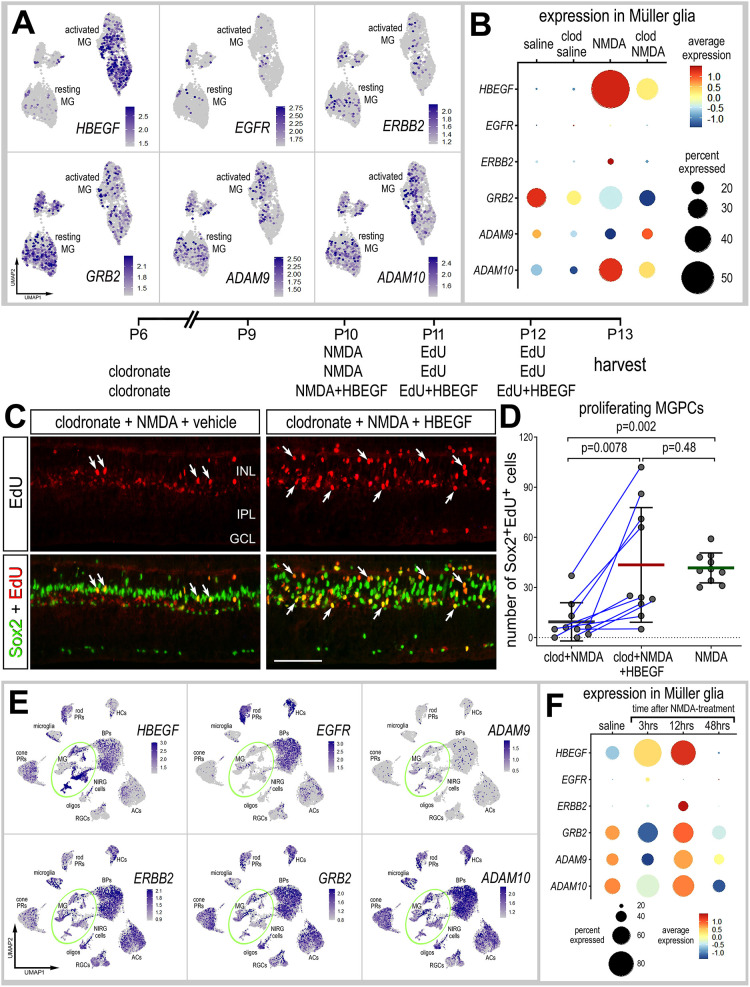
**Activation of HBEGF rescues the deficit in MGPC formation in damaged retinas missing microglia.** (A,B,E,F) Patterns and levels of expression of EGF-related genes in scRNA-seq libraries of retinas treated with saline±clodronate and NMDA±clodronate (A,B), or different times after NMDA treatment (E,F). UMAP heatmap plots (feature plots) illustrate patterns and levels of expression for EGF-related genes in MG only (A) or across all types of retinal neurons and glia (E). UMAP heatmap plots (E) and dotplot (F) illustrate patterns, percent expressed and levels of expression for HBEGF-related genes in retinal neurons and glia in control retinas and retinas at 3, 12 and 48 h after NMDA treatment. The dot plots in B and F illustrate expression levels (heatmap) and percent expressed (dot size) for different genes in MG. (C,D) Retinal sections labeled for EdU accumulation (red; C) and with antibodies to Sox2 (green; C). Arrows indicate the nuclei of MG. Scale bar: 50 µm. The graph in D illustrates the mean (±s.d.) and each dot represents one biological replicate. Significance of difference (*P*-values) was determined using an unpaired *t*-test with Bonferroni correction (D).

MG significantly upregulate HBEGF after NMDA treatment, and injections of HBEGF stimulate the proliferation of MGPCs (Todd et al., 2015; Fig. 1H). We found that the upregulation of *HBEGF* by MG was greatly diminished when microglia were absent (Figs 1H and 5A,B; Tables S2, S5). Levels of the EGF receptors *ERBB2* and *GRB2* were not significantly affected by the presence of microglia in normal or damaged retinas (Fig. 5A,B; Tables S2, S5). Although *EGFR* was expressed at very low levels, there was a significant increase in MG undamaged retinas when microglia were ablated (Fig. 5A,B; Tables S1, S5). We next probed for patterns of expression of *ADAM9* and *ADAM10*, enzymes known to process HBEGF in the extracellular space. ADAM9 is involved in tissue plasminogen activator (TPA)-induced ectodomain shedding of membrane-tethered HBEGF. ADAM10 proteolytically releases cell-surface proteins, including HBEGF, ephrin A2, CD44 and CDH2 (Jouannet et al., 2016; Lemjabbar and Basbaum, 2002; Seegar et al., 2017). *ADAM9* was expressed by resting MG and was downregulated at 24 h after NMDA treatment and when the microglia were ablated in undamaged retinas, but was upregulated by MG at 24 h after NMDA when microglia were ablated (Fig. 5A,B). *ADAM10* was upregulated by MG at 24 h after NMDA treatment, and this upregulation was not significantly affected when microglia were ablated (Fig. 5A,B; Tables S2, S5).

We tested whether HBEGF rescued the deficit of MGPC proliferation in damaged retinas missing reactive microglia. HBEGF is known to stimulate the formation of proliferating MGPCs in the retinas of fish (Wan et al., 2012), chicks and mice (Todd et al., 2015). We found that injections of HBEGF with and following NMDA treatment significantly increased numbers of proliferating MGPCs in retinas missing microglia (Fig. 5C,D). Consistent with this observation, we found that the Sox2-positive nuclei of MG migrated away from the center of the INL (Fig. 5C,D).

To better understand the time course of changes in expression, we probed for expression levels of EGF-related genes in the retina and MG in scRNA-seq libraries generated shortly (3 or 12 h) after NMDA treatment. We found that *HBEGF* was rapidly upregulated within 3 h of NMDA treatment (Fig. 5E,F; Tables S4, S5). Levels of *HBEGF* were further increased at 12 h and were absent by 48 h after NMDA treatment (Fig. 5E,F). We next probed for expression of EGF receptors. We found that *ERBB2* and *GRB2* were widely expressed by different types of retinal cells, whereas *EGFR* was predominantly expressed by bipolar cells, rod photoreceptors and horizontal cells (Fig. 5E). In MG, levels of *GRB2* and *ERBB2* were significantly changed following damage, whereas levels of *EGFR* were unaffected (Fig. 5E; Tables S4, S5). *ADAM9* was not widely expressed by different types of retinal neurons, but was expressed by resting MG and was downregulated at 3 h, up at 12 h and back down by 48 h after NMDA treatment (Fig. 5E,F; Tables S4, S5). By comparison, *ADAM10* was widely expressed by different types of retinal neurons and glia (Fig. 5E). Similar to patterns of expression of *ADAM9* in MG, *ADAM10* was downregulated at 3 h, up at 12 h and back down by 48 h after NMDA treatment (Fig. 5E; Tables S4, S5). Collectively, these findings are consistent with the notion that HBEGF-mediated autocrine signaling in MG is a key component of the early processes of reprogramming into MGPCs, similar to that described for regeneration in the fish retina (Wan et al., 2012).

### Inhibition of Smad3 and the formation of MGPCs in the absence of microglia

**Fig. 6.**
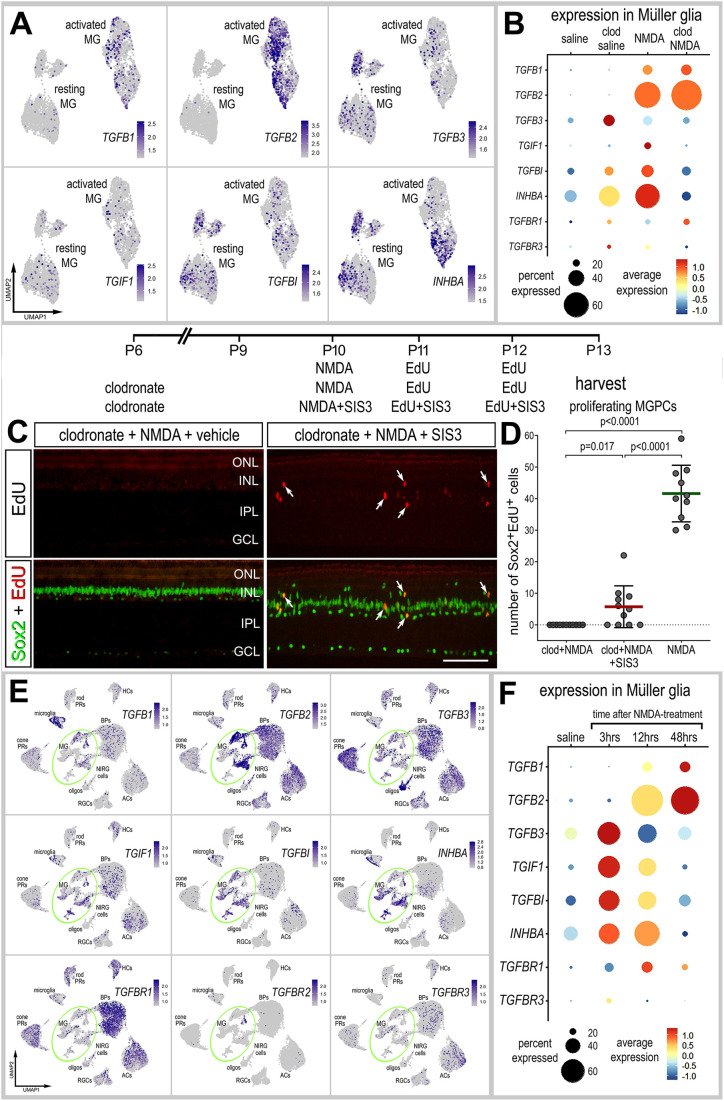
**Activation of TGFβ/Smad3 signaling partially rescues the deficit in MGPC formation in damaged retinas missing microglia.** (A,B,E,F) Patterns and levels of expression of TGFβ-related genes in scRNA-seq libraries of retinas treated with saline±clodronate and NMDA±clodronate (A,B), or different times after NMDA treatment (E,F). UMAP heatmap plots illustrate patterns and levels of expression for TGFβ-related genes in MG only (A) or across all types of retinal neurons and glia (E). UMAP heatmap plots (E) and dotplot (F) illustrate patterns, percent expressed and levels of expression for TGFβ-related genes in retinal neurons and glia in control retinas and retinas at 3, 12 and 48 h after NMDA treatment. The dot plots in B and F illustrate expression levels (heatmap) and percent expressed (dot size) for different genes in MG. (C,D) Retinal sections labeled for EdU accumulation (red; C) and with antibodies to Sox2 (green; C). Arrows indicate the nuclei of MG. Scale bar: 50 µm. The graph in D illustrates the mean (±s.d.) and each dot represents one biological replicate. Significance of difference (*P*-values) was determined using Mann–Whitney *U*-test with Bonferroni correction (D).

Signaling through TGFβ2 and Smad2/3 has been shown to act, in opposition to BMP and Smad1/5/8, to suppress the formation of proliferating MGPCs (Todd et al., 2017). Accordingly, we probed for changes in expression levels of TGFβ-related genes in MG in normal and damaged retinas, with and without microglia. Although *TGFB1* and *TGFB2* were significantly increased in MG in damaged retinas, the ablation of microglia did not significantly affect levels of expression (Fig. 6A,B; Tables S2, S5). By comparison, the ablation of microglia caused MG to significantly upregulate *TGFB3* and *INHBA* (Fig. 6A,B; Tables S2, S5). We next probed for genes related to TGFβ signaling. We found that *TGIF1* (TGFβ induced factor homeobox 1) is a transcriptional co-repressor of Smad2 that regulates TGFβ signaling (Guca et al., 2018). TGFBI (transforming growth factor beta induced) is induced by TGFβ and acts to inhibit cell adhesion (LeBaron et al., 1995; Skonier et al., 1994). *INHBA* encodes a member of the TGFβ superfamily of proteins; the preproprotein is proteolytically processed to generate a subunit of the dimeric activin and inhibin protein complexes (Antenos et al., 2008; Ling et al., 1986). The ablation of microglia from damaged retinas resulted in MG maintaining high levels *TGFB2/3*, but significantly downregulated the inhibitors *TGIF1* and *INHBA*; changes in levels of *TGFBI* were not significant (Fig. 6A,B; Tables S2, S5). There were no significant changes in levels of TGFβ receptors (Fig. 6A,B; Tables S2, S5). Collectively, these findings suggest that in damaged retinas missing microglia there may be increased TGFβ/Smad autocrine signaling among MG that suppresses the formation of MGPCs. Accordingly, we tested whether inhibition of TGFβ/Smad signaling rescued the deficit in MGPC proliferation in damaged retinas missing microglia. Injections of a Smad3 antagonist (SIS3) to NMDA-damaged retinas missing microglia resulted in a small, but significant increase in numbers of proliferating MGPCs (Fig. 6C,D). This increase in MGPC proliferation was significantly less than that seen in retinas treated with NMDA alone (Fig. 6D). Consistent with this observation, we found that there was relatively little delamination of Sox2-positive nuclei of MG away from the center of the INL in damaged microglia-depleted retinas treated with the Smad3 antagonist (Fig. 6C).

To better understand the time course of changes in expression, we probed for expression levels of TGFβ-related genes in the retina and MG in scRNA-seq libraries generated shortly (3 or 12 h) after NMDA treatment. *TGFB1* was predominantly expressed by microglia (Fig. 6E). By comparison, *TGFB2* was predominantly expressed by activated MG, bipolar cells and amacrine cells (Fig. 6E,F). *TGFB3* was widely expressed by cone photoreceptors and oligodendrocytes, and showed scattered expression across all other types of retina neurons and glia (Fig. 6E). In MG, specifically, *TGFB1* and *TGFB2* were significantly upregulated at 12 and 48 h after NMDA treatment, with *TGFB2* being expressed by most MG (Fig. 6E; Tables S4, S5), whereas *TGFB3* was significantly upregulated in MG at 3 h after NMDA treatment and downregulated thereafter (Fig. 6E; Tables S4, S5). *TGIF1*, *TGFBI* and *INHBA* were predominantly expressed in MG (Fig. 6E,F). Similar to patterns of expression of *TGFB3*, levels of these factors were low in resting MG, upregulated within 3 h, remained elevated at 12 h, and downregulated by 48 h after NMDA treatment (Fig. 6E; Tables S4, S5). The TGFβ receptors *TGFBR1* and *TGFBR3* were expressed by different types of retinal neurons and glia, whereas *TGFBR2* was not widely expressed in the retina (Fig. 6E). *TGFBR1* was significantly upregulated by a relatively small percentage of MG at 12 h after treatment, whereas *TGFBR3* was not expressed by MG or upregulated by MG in damaged retinas (Fig. 6E; Tables S4, S5). Collectively, these findings indicate that increased autocrine TGFβ/SMAD signaling may occur in the absence of microglia because of a loss of upregulation of inhibitors to this pathway, and that inhibition of SMAD3 partially rescues the deficit in MGPC formation.

### Activation of RARα and the formation of MGPCs in retinas missing microglia

**Fig. 7.**
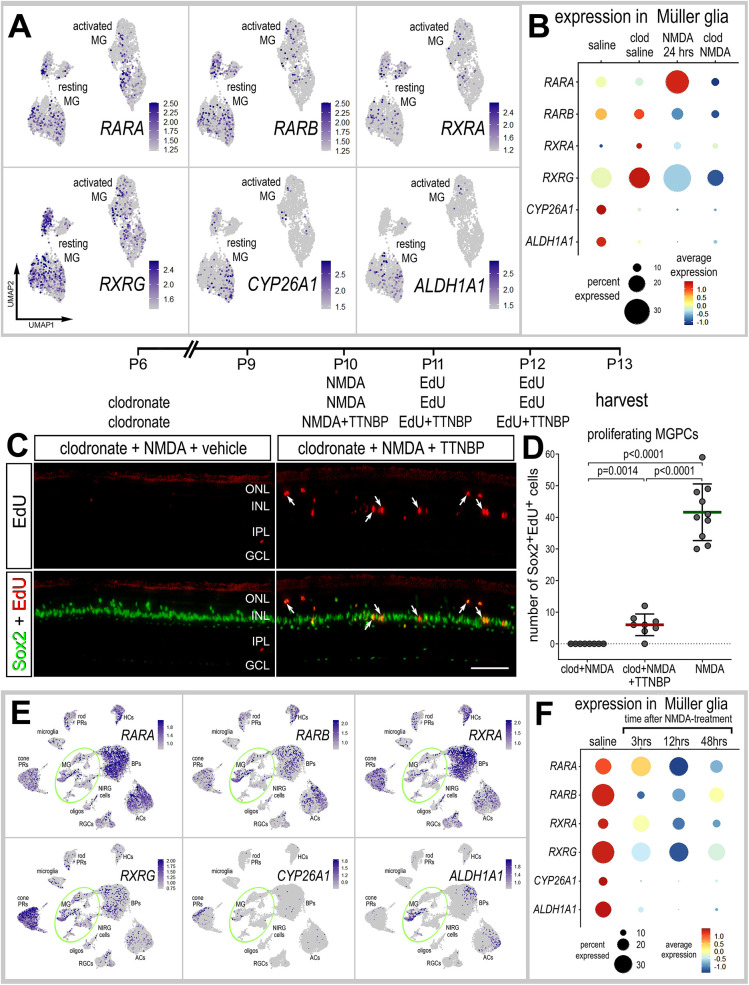
**Inhibition of retinoic acid signaling partially rescues the deficit in MGPC formation in damaged retinas missing microglia.** (A,B,E,F) Patterns and levels of expression of retinoic acid- related genes in scRNA-seq libraries of retinas treated with saline±clodronate and NMDA±clodronate (A,B), or different times after NMDA (E,F). UMAP heatmap plots (feature plots) illustrate patterns and levels of expression for retinoic acid-related genes in MG only (A) or across all types of retinal neurons and glia (E). UMAP heatmap plots (E) and dotplot (F) illustrate patterns, percent expressed and levels of expression for RAR-related genes in retinal neurons and glia in control retinas and retinas at 3, 12 and 48 h after NMDA treatment. The dot plots in B and F illustrate expression levels (heatmap) and percent expressed (dot size) for different genes in MG. (C,D) Retinal sections were labeled for EdU accumulation (red; C) and immunolabeled for Sox2 (green; C). Arrows indicate the nuclei of MG. Scale bar: 50 µm. The graph in D illustrates the mean (±s.d.) and each dot represents one biological replicate. Significance of difference (*P*-values) was determined using a Mann–Whitney *U*-test with Bonferroni correction (D).

Signaling through retinoic acid receptors is known to stimulate the proliferation of MGPCs and increase the number of progeny that differentiate as neurons (Todd et al., 2018). One of the key receptors of retinoic acid (RA) is encoded by *RARA*, which is highly upregulated by MG in damaged retinas, but fails to become upregulated when microglia are absent (Figs 1G,H and 7A,B; Tables S2, S5). By comparison, *RARB* is downregulated by MG in damaged retinas, but is upregulated in resting MG when microglia are ablated (Fig. 7A,B; Tables S2, S5). Similarly, *CYP26A1*, encoding an enzyme that inactivates RA via oxidation, and *ALDH1A1*, encoding an enzyme involved in the synthesis of RA, are downregulated in MG when microglia are ablated and in damaged retinas (Fig. 7A,B; Tables S2, S5). *ALDH1A2* was not detected at significant levels in the chick retina (Fig. S4E). Injections of a RAR agonist, TTNBP, to NMDA-damaged retinas missing microglia resulted in a small, but significant increase in numbers of proliferating MGPCs labeled for EdU and Sox2 (Fig. 7C,D). Consistent with this observation, we found that there was relatively little delamination of Sox2-positive nuclei of MG away from the center of the INL in damaged microglia-depleted retinas treated with RAR agonist (Fig. 7C). It is worth noting that intra-ocular injections of TTNBP compromised the survival of about 50% the chicks; we have not observed this effect for dozens of other compounds that we have applied to the eye of chicks over the past 25 years.

To better understand the time course of changes in expression, we probed for expression levels of RAR-related genes in the retina and MG in scRNA-seq libraries generated shortly (3 or 12 h) after NMDA treatment. *RARA*, *RARB* and *RXRA* were expressed by most types of neurons and glia, whereas *RXRG* was predominantly expressed by cone photoreceptors (Fig. 7E). By comparison, *CYP26A1* was expressed by some cone photoreceptor and *ALDH1A1* was expressed by some MG, bipolar cells and amacrine cells (Fig. 7E). In MG, RAR-related genes, with the exception of *RXRA*, were significantly and rapidly downregulated following NMDA treatment (Fig. 7E; Tables S4, S5).

## DISCUSSION

**Fig. 8.**
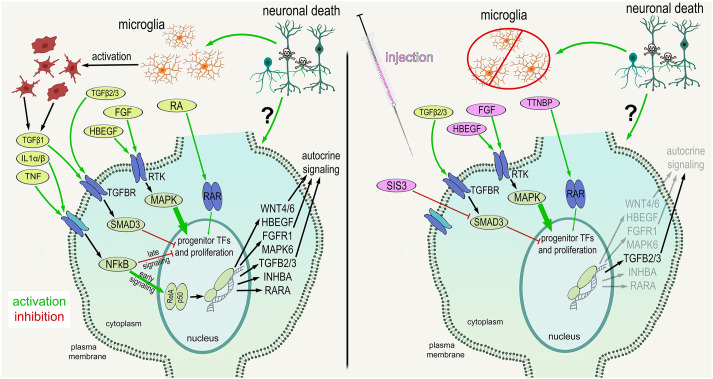
**Schematic summarizing the factors and cell signaling pathways downstream of activated microglia that are key to the formation of MGPCs.** The schematic summarizes some of the ligands, receptors and pathways that are activated in MG in damaged retinas. The factors that rescue the deficit in MGPC proliferation that occurs in damaged retinas missing microglia are illustrated in the panel to the right.

In this study, we investigate how the depletion of microglia from the retina impacts MG and the ability of MG to reprogram into proliferating progenitor-like cells. We found significant transcriptomic changes in resting and activated MG in normal and damaged retinas when the microglia were absent. We identified significant changes in gene modules related to different signaling pathways that have been implicated in regulating the formation of MGPCs. These gene modules included Wnt signaling, Notch signaling, fatty acid binding, BMP signaling and retinoic acid signaling, all of which have been implicated in stimulating the proliferation of MGPCs in damaged chick retinas (Campbell et al., 2022; Gallina et al., 2015; Ghai et al., 2010; Hayes et al., 2007; Todd et al., 2017, 2018). Intra-ocular injections of HBEGF or FGF2 completely rescued the deficit in MGPC formation in damaged retinas missing microglia, whereas injections of RARα agonist or Smad3 antagonist partially rescued this deficit (Fig. 8).

It is possible that rapid activation of signaling pathways is required to drive the process of MG reprogramming, and these pathways require rapid activation of signals from microglia. In the mouse retina, for example, microglia upregulate IL1β, IL1α and TNFα within 3 h of NMDA treatment (Todd et al., 2019), and these pro-inflammatory cytokines activate NFκB signaling in MG (Palazzo et al., 2022, 2023). We have recently reported, in the chick retina, that activation of NFκB signaling with prostratin or injection of a TNF ligand (TNFSF15) rescues the proliferation of MGPCs in damaged retinas in which the microglia have been ablated (Palazzo et al., 2020). Prostratin activates NFκB by stimulating phosphorylation and degradation of IκBα in an IKK-dependent manner (Williams et al., 2004). These data indicate that microglia rapidly respond to neuronal damage to produce pro-inflammatory cytokines to stimulate the first steps in MG activation/reactivity that are required for the formation of MGPCs. In fish and chick retinas, MG undergo activation prior to becoming proliferating MGPCs (Hoang et al., 2020). Interestingly, MG-specific conditional knock out of *Ikkb* causes a significant downregulation of MAPK-signaling gene modules in damaged mouse retinas, suggesting that activation of MAPK gene networks are downstream of NFκB in activated MG (Palazzo et al., 2020). Consistent with these findings, FGF2 does not activate NFκB signaling in mouse retinas (Palazzo et al., 2023), suggesting that activation of MAPK signaling in MG may be downstream or independent of NFκB. Collectively, these findings suggest that pro-inflammatory cytokines are among the early signals provided by microglia to ‘kick start’ the process of MG reprogramming (Fig. 8).

### GSK3β inhibitors fail to stimulate the proliferation of MGPCs

Activation of Wnt signaling is known to stimulate the proliferation of MGPCs in damaged retinas of zebrafish (Ramachandran et al., 2011), chick (Gallina et al., 2015) and rodents (Osakada et al., 2007; Yao et al., 2016). However, inhibition of GSK3β, which normally occurs downstream of activated Wnt receptors, did not stimulate the proliferation of MGPCs in damaged retinas missing microglia. These results raise the question of why GSK3β inhibition did not stimulate MGPC proliferation in the absence of microglia. It is possible that Wnt signaling is activated later in the process of reprograming and first requires activation of MAPK signaling initiate the process. Consistent with this notion, HBEGF and components of the MAPK pathway are upregulated within 3 h of NMDA treatment, whereas *WNT4* and *WNT6* are not upregulated until 12 h after treatment. Alternatively, the targets of GSK3β inhibitors may be downregulated by MG when microglia are ablated. However, levels of β-catenin and GSK3β are significantly upregulated by MG by in NMDA-damaged retinas, and this upregulation is not affected by the ablation of microglia (Fig. S4F). Thus, the targets for GSK3β inhibitors were in place in MG to elicit a response, and this response appears to be restricted to cellular migration.

The delamination of MG nuclei away from the middle of the INL is associated with the proliferation of MGPCs (Fischer and Reh, 2001, 2003; Fischer et al., 2004). The vast majority of proliferating MGPCs in M-phase are displaced away from the middle of the INL and are usually found in the proximal INL or distal INL and outer nuclear layer (Fischer and Reh, 2001, 2003). Further, the formation of MGPCs is associated with delamination of MG nuclei away from the middle of the INL (Fischer and Bongini, 2010; Gallina et al., 2014). For example, in the current study we observed modest increases in MGPC proliferation in damaged retinas missing microglia treated with RAR agonist or Smad3 inhibitor, and this modest increase in proliferation was associated with modest delamination of MG nuclei. However, we found widespread delamination of MG nuclei away from the middle of the INL in response to GSK3β inhibitors in damaged retinas missing microglia, without evidence of proliferation. Similarly, activation of Hedgehog signaling with a smoothened agonist and IGF1 stimulates the delamination of MG nuclei without proliferation (Todd and Fischer, 2015). Further, application of MMP2/9 inhibitor with FGF2 stimulates the delamination of MG nuclei (Campbell et al., 2019). By comparison, inhibition of S-adenosyl homocysteine hydrolase and histone methylation potently suppresses the delamination of MG nuclei and proliferation in retinas treated with NMDA or insulin+FGF2 (Campbell et al., 2023). Thus, these observations indicate that migration can be de-coupled from proliferation, but proliferation of MGPCs is always associated with nuclear migration.

### Effects of FGF2 and insulin+FGF2

We have previously reported that FGF2 alone or insulin+FGF2 stimulate the formation of proliferating MGPCs in undamaged retinas (Fischer et al., 2002, 2014). However, these treatments fail to stimulate the formation of MGPCs in undamaged retinas when the microglia have been ablated (Fischer et al., 2014). Interestingly, we found that intra-ocular injection of FGF2 stimulated the formation of MGPCs in damaged retinas missing microglia. Collectively, these findings suggest that damaged neurons in retinas missing microglia provide signals that permit or act synergistically with FGF2 to drive the reprogramming of MG into proliferating MGPCs. The identity of signals provided by NMDA-damaged retinal neurons remains unknown (Fig. 8).

### Amplitude of effects on MGPC rescue

HBEGF and FGF2 administration led to robust rescue effects upon stimulating the proliferation of MGPCs in damaged retinas missing microglia. Consistent with these observations, HBEGF and FGF2 potently stimulate the proliferation of MGPCs in damaged retinas (Fischer et al., 2014; Todd et al., 2015). Similarly, inhibitors of MAPK signaling and FGF receptor inhibitors potently suppress the proliferation of MGPCs in damaged retinas (Fischer et al., 2009a,b). By comparison, the Smad3 inhibitor and RAR agonist had relatively small effects upon stimulating the proliferation of MGPCs in damaged retinas missing microglia. Alternatively, it is possible that the failed upregulation of *RARA* by MG in retinas missing microglia was not efficiently rescued because the levels of *RARA* remained low, leaving the agonist with few targets to act upon. Another interpretation is that retinoic acid signaling ‘fine-tunes’ the proliferating stimulating pathways, and that TGFβ/Smad3 acts to suppress the proliferation response (Fig. 8).

It is unlikely that levels of cell death impacted the outcomes of these studies because the ablation of microglia, or application of growth factors or small molecules inhibitors used in this study have been shown to either reduce or have no effect on the numbers of dying cells (Fischer et al., 2009a, 2015; Gallina et al., 2015; Todd et al., 2017, 2018). It is expected that reduced levels of cell death would reduce numbers of proliferating MGPCs. However, these growth factors and small molecule inhibitors all increased numbers of proliferating MGPCs, with the exception of the cocktail of GSK3β inhibitors.

### Conclusions

We conclude that quiescent and reactive microglia have a significant impact upon the transcriptomic profile of MG. The absence of reactive microglia from damaged retinas results in the failure of activated MG to upregulate many different networks of genes related to different pathways known to influence the reprogramming of MG into proliferating MGPCs. Many of these genes are rapidly up- or downregulated by MG in response to acute retinal damage. We conclude that signals produced by reactive microglia in damaged retinas normally stimulate MG to upregulate cell signaling through HBEGF, FGF/MAPK and RAR, and downregulate inhibitory signaling through TGFβ/Smad3.

## MATERIALS AND METHODS

### Animals

The animal use in these experiments was approved in accordance with the guidelines established by the National Institutes of Health and IACUC at The Ohio State University. Newly hatched postnatal day (P) 0 wild-type leghorn chicks (*Gallus domesticus*) were obtained from Meyer Hatchery (Polk, OH, USA). Post-hatch chicks were maintained in a regular diurnal cycle of 12 h light (08:00-20:00 h), 12 h dark. Chicks were housed in stainless-steel brooders at 25°C and received water and Purina™ chick starter *ad libitum*.

### Intra-ocular injections

Chicks were anesthetized with 2.5% isoflurane mixed with oxygen from a non-rebreathing vaporizer. The technical procedures for intra-ocular injections were performed as previously described (Fischer et al., 1998). With all injection paradigms, both pharmacological and vehicle treatments were administered to the right and left eye, respectively. Compounds were injected in 20 µl sterile saline with 0.05 mg/ml bovine serum albumin added as a carrier. Compounds used were: (1) NMDA (38.5 nmol or 154 µg/dose; Millipore Sigma); (2) FGF2 (250 ng/dose; R&D Systems); (3) HBEGF (500 ng/dose, R&D Systems); (4) a cocktail of three different GSK3β inhibitors (ABC) – 1-azakenpaullone (500 ng/dose; Selleck Chemicals), BIO (500 ng/dose; R&D Systems) and CHIR 99021 (500 ng/dose; R&D Systems); (5) the Smad3 inhibitor SIS3 (2 µg/dose; Sigma-Aldrich), and (6) the retinoic acid receptor agonist TTNBP {2 µg/dose; 4-[(*E*)-2-(5,6,7,8-tetrahydro-5,5,8,8-tetramethyl-2-naphthalenyl)-1-propenyl]benzoic acid; Tocris}. EdU (Thermo Fisher Scientific) was injected into the vitreous chamber to label proliferating cells. Injection paradigms are included in each figure.

### Preparation of clodronate liposomes

The preparation of clodronate liposomes was similar to previous descriptions (van Rooijen, 1992; Zelinka et al., 2012). In brief, 50 ng cholesterol and 8 mg egg lecithin (L-α-phosphatidyl-DL-glycerol sodium salt; Sigma-Aldrich) were dissolved in chloroform in a round-bottom flask. The solution was evaporated under nitrogen to leave a liposome residue. Then, 158 mg dichloro-methylene diphosphonate (clodronate; Sigma-Aldrich) in sterile PBS was added and mixed. Clodronate encapsulation was facilitated by sonication at 42,000 Hz for 5 min. Liposomes were centrifuged at 10,000 ***g*** for 15 min and re-suspended in 150 ml sterile PBS. We titered doses for each preparation of liposome to levels at which >99% of microglia/macrophages were ablated at 2 days after intra-ocular injection.

### scRNA-seq

Retinas were dissociated in a 0.25% papain solution in Hank's balanced salt solution (pH 7.4) (Worthington Biochemicals), for 20 min, and suspensions were triturated. The dissociated cells were passed through a sterile 70 µm filter to remove large particulate debris. Cells were assessed for viability (Countess II; Invitrogen) and diluted to 700 cells/µl. Each library was prepared for a target of 10,000 cells per sample. The cell suspension and Chromium Single Cell 3′ V2 or V3 reagents (10x Genomics) were loaded onto chips to capture individual cells with individual gel beads in emulsion (GEMs) using the 10x Chromium Cell Controller. cDNA and library amplification and for optimal signal was 12 and 10 cycles, respectively. Sequencing was conducted on Illumina HiSeq2500 (Genetic Resources Core Facility, Johns Hopkins University, MD, USA), or Novaseq6000 (Novogene) using 150 paired-end reads. Fasta sequence files were de-multiplexed, aligned, and annotated using the chick ENSMBL database (GRCg6a, Ensembl release 94) using 10x Cell Ranger v3.1.0 (Figs 1, 3-7) or v7.0.1 (Fig. 2). Gene expression was counted using UMI bar codes and gene–cell matrices were constructed. Using Seurat v4.0.0-4.3.0, UMAP plots were generated from aggregates of multiple scRNA-seq libraries (Butler et al., 2018; Satija et al., 2015). In short, Seurat objects were created from Cell Ranger filtered matrix files. Objects were merged, filtered (<200 genes/cell, >2500 genes/cell, >5% mitochondrial genes) and normalized (logNormalize, default scale factor), highly variable features were identified (selection method=’vst’, nfeatures≥2000), data scaled using default settings, linear dimensions reduced (principal component analysis), elbow plots established to determine dimensions with >2 s.d., cell clusters with appropriate numbers of dimensions (FindNeighbors using dimensions from elbow plots, FindClusters with default resolution), and UMAP ordering of cells established using at least ten dimensions. Seurat was used to construct gene lists for DEGs, violin/scatter plots and dot plots. Significance of difference was determined using a Wilcoxon Rank Sum test with Bonferroni correction. SingleCellSignalR v1.10.0 was used to assess potential LR interactions between cells within scRNA-seq datasets (Cabello-Aguilar et al., 2020). LR interaction networks were visualized using Cytoscape (Shannon et al., 2003). Genes that were used to identify different types of retinal cells included the following: (1) Müller glia: *GLUL*, *VIM*, *SLC1A3*, *RLBP1*; (2) MGPCs: *PCNA*, *CDK1*, *TOP2A*, *ASCL1*; (3) microglia and macrophages: *C1QA*, *C1QB*, *CCL4*, *CSF1R*, *SLC35G2*; (4) ganglion cells: *THY1*, *POU4F2*, *RBPMS2*, *NEFL*, *NEFM*; (5) amacrine cells: *GAD1*, *CALB2*, *TFAP2A*; (6) horizontal cells: *PROX1*, *CALB2*, *NTRK1*; (7) bipolar cells: *VSX1*, *OTX2*, *GRIK1*, *GABRA1*; (7) cone photoreceptors: *CALB1*, *GNAT2*, *OPN1LW*; and (8) rod photoreceptors: *RHO*, *NR2E3*, *ARR3*.

GO enrichment analysis was performed using ShinyGO V0.72 (http://bioinformatics.sdstate.edu/go/). We selected significant relevant terms for biological function and molecular function GO categories, and for KEGG pathways. Adjustment for multiple hypothesis testing is performed using ShinyGO by normalizing the enrichment score for each gene set to account for the size of the set, to produce a normalized enrichment score (NES). The proportion of false positives is controlled by calculating the false discovery rate (FDR) corresponding to each NES. The FDR enrichment *P*-value is calculated as the probability that a gene set with a given NES represents a false-positive finding; the probability is computed by comparing the tails of the observed and null distributions for the NES (Ge et al., 2020).

### Fixation, sectioning and immunocytochemistry

Retinas were fixed, sectioned and immunolabeled as described previously (Fischer et al., 2001; Ghai et al., 2009; Ritchey et al., 2010). Antibody dilutions and commercial sources are described in Table S7. Sections incubated with secondary antibodies alone were devoid of fluorescence, indicating that the observed labeling was not due to off-target labeling of secondary antibodies or tissue auto-fluorescence. Secondary antibodies utilized were: donkey anti-goat Alexa Fluor 488/568, goat anti-rabbit Alexa Fluor 488/568/647, goat anti-mouse Alexa Fluor 488/568/647 and goat anti-rat Alexa Fluor 488 (Life Technologies), diluted to 1:1000 in PBS and 0.2% Triton X-100.

### Labeling for EdU

For the detection of EdU, immunolabeled sections were fixed in 4% formaldehyde in 0.1 M PBS pH 7.4 for 5 min at room temperature. Samples were washed for 5 min with PBS, permeabilized with 0.5% Triton X-100 in PBS for 1 min at room temperature, and washed twice for 5 min in PBS. Sections were incubated for 30 min at room temperature in 100 mM Tris, 8 mM CuSO_4_ and 100 mM ascorbic acid in dH_2_O. Alexa Fluor 568 Azide (Thermo Fisher Scientific) was added to the buffer at a 1:100 dilution.

### Imaging, measurements, cell counts and statistics

Digital photomicroscopy was performed using a Leica DM5000B microscope with epifluorescence and Leica DC500 camera. Confocal images were obtained with a Leica SP8. Representative images were modified to optimized for color, brightness and contrast using Adobe Photoshop 2023. In proliferation assays, EdU-positive cells were counted with a fixed area/region of retina and average numbers of Sox2 and EdU co-labeled cells were determined. The retinal region selected for investigation was standardized between treatment and control groups.

Prism 10 (GraphPad) was used for statistical analyses. A Levene's test was used to determine whether data from control and treatment groups had equal variance. For treatment groups for which the Levene's test indicated unequal variance, a Wilcoxon rank-sum test was used. For statistical evaluation of parametric data, a two-tailed, paired *t*-test was used to account for intra-individual variability whereby each biological sample served as its own control (left eye, control; right eye, treated). For multivariate analysis across more than two treatments, an ANOVA with Tukey's post-hoc test was performed to evaluate significant differences between multiple groups.

## Supplementary Material

10.1242/develop.202070_sup1 Supplementary information

Table S1. DEGs in MG in retinas treated with saline (pct.1) vs saline + clodronate-liposomes (pct.2). Negative avg log2FC represents an increase and positive avg log2FC represents a decrease.

Table S2. DEGs in MG in retinas treated with NMDA (pct.1) vs NMDA + clodronate-liposomes (pct.2). Negative avg log2FC represents an increase and positive avg log2FC represents a decrease.

Table S3. DEGs in MG in retinas treated with saline (pct.1) vs NMDA (pct.2). Negative avg log2FC represents an increase and positive avg log2FC represents a decrease.

Table S4. DEGs in MG in retinas treated with saline or NMDA and retinas harvested at 3, 12 or 48 hours later. DEGs were identified for resting MG vs 3+12+48hr NMDA MG, resting MG vs 3hr NMDA MG, resting MG vs 12hr NMDA MG, and resting MG vs 48hr NMDA MG. Negative avg log2FC represents an increase and positive avg log2FC represents a decrease

Table S6. Lists of significant autocrine LR-interactions in MG and Venn diagram lists for figure 2.

## References

[DEV202070C1] Antenos, M., Zhu, J., Jetly, N. M. and Woodruff, T. K. (2008). An activin/furin regulatory loop modulates the processing and secretion of inhibin α- and βB-subunit dimers in pituitary gonadotrope cells. *J. Biol. Chem.* 283, 33059-33068. 10.1074/jbc.M804190200 18826955 PMC2586270

[DEV202070C2] Bernardos, R. L., Barthel, L. K., Meyers, J. R. and Raymond, P. A. (2007). Late-stage neuronal progenitors in the retina are radial Müller glia that function as retinal stem cells. *J. Neurosci.* 27, 7028-7040. 10.1523/JNEUROSCI.1624-07.2007 17596452 PMC6672216

[DEV202070C3] Butler, A., Hoffman, P., Smibert, P., Papalexi, E. and Satija, R. (2018). Integrating single-cell transcriptomic data across different conditions, technologies, and species. *Nat. Biotechnol.* 36, 411-420. 10.1038/nbt.4096 29608179 PMC6700744

[DEV202070C4] Cabello-Aguilar, S., Alame, M., Kon-Sun-Tack, F., Fau, C., Lacroix, M. and Colinge, J. (2020). SingleCellSignalR: inference of intercellular networks from single-cell transcriptomics. *Nucleic Acids Res.* 48, e55. 10.1093/nar/gkaa183 32196115 PMC7261168

[DEV202070C5] Campbell, W. A., Deshmukh, A., Blum, S., Todd, L., Mendonca, N., Weist, J., Zent, J., Hoang, T. V., Blackshaw, S., Leight, J. et al. (2019). Matrix-metalloproteinase expression and gelatinase activity in the avian retina and their influence on Müller glia proliferation. *Exp. Neurol.* 320, 112984. 10.1016/j.expneurol.2019.112984 31251936 PMC6716156

[DEV202070C6] Campbell, W. A., Fritsch-Kelleher, A., Palazzo, I., Hoang, T., Blackshaw, S. and Fischer, A. J. (2021a). Midkine is neuroprotective and influences glial reactivity and the formation of Müller glia-derived progenitor cells in chick and mouse retinas. *Glia* 69, 1515-1539. 10.1002/glia.23976 33569849 PMC8194292

[DEV202070C7] Campbell, W. A., Blum, S., Reske, A., Hoang, T., Blackshaw, S. and Fischer, A. J. (2021b). Cannabinoid signaling promotes the de-differentiation and proliferation of Müller glia-derived progenitor cells. *Glia* 69, 2503-2521. 10.1002/glia.24056 34231253 PMC8373766

[DEV202070C8] Campbell, W. A., Tangeman, A., El-Hodiri, H. M., Hawthorn, E. C., Hathoot, M., Blum, S., Hoang, T., Blackshaw, S. and Fischer, A. J. (2022). Fatty acid-binding proteins and fatty acid synthase influence glial reactivity and promote the formation of Müller glia-derived progenitor cells in the chick retina. *Development* 149, dev200127. 10.1242/dev.200127 35132991 PMC8959147

[DEV202070C9] Campbell, W. A., El-Hodiri, H. M., Torres, D., Hawthorn, E. C., Kelly, L. E., Volkov, L., Akanonu, D. and Fischer, A. J. (2023). Chromatin access regulates the formation of Müller glia-derived progenitor cells in the retina. *Glia* 71, 1729-1754. 10.1002/glia.24366 36971459 PMC11335016

[DEV202070C10] El-Hodiri, H. M., Campbell, W. A., Kelly, L. E., Hawthorn, E. C., Schwartz, M., Jalligampala, A., Mccall, M. A., Meyer, K. and Fischer, A. J. (2022). Nuclear Factor I in neurons, glia and during the formation of Müller glia-derived progenitor cells in avian, porcine and primate retinas. *J. Comp. Neurol.* 530, 1213-1230. 10.1002/cne.25270 34729776 PMC8969175

[DEV202070C11] Fausett, B. V. and Goldman, D. (2006). A role for alpha1 tubulin-expressing Müller glia in regeneration of the injured zebrafish retina. *J. Neurosci.* 26, 6303-6313. 10.1523/JNEUROSCI.0332-06.2006 16763038 PMC6675181

[DEV202070C12] Fausett, B. V., Gumerson, J. D. and Goldman, D. (2008). The proneural basic helix-loop-helix gene ascl1a is required for retina regeneration. *J. Neurosci.* 28, 1109-1117. 10.1523/JNEUROSCI.4853-07.2008 18234889 PMC2800945

[DEV202070C13] Fischer, A. J. and Bongini, R. (2010). Turning Müller glia into neural progenitors in the retina. *Mol. Neurobiol.* 42, 199-209. 10.1007/s12035-010-8152-2 21088932

[DEV202070C14] Fischer, A. J. and Reh, T. A. (2001). Müller glia are a potential source of neural regeneration in the postnatal chicken retina. *Nat. Neurosci.* 4, 247-252. 10.1038/85090 11224540

[DEV202070C15] Fischer, A. J. and Reh, T. A. (2003). Potential of Müller glia to become neurogenic retinal progenitor cells. *Glia* 43, 70-76. 10.1002/glia.10218 12761869

[DEV202070C16] Fischer, A. J., Seltner, R. L. P., Poon, J. and Stell, W. K. (1998). Immunocytochemical characterization of quisqualic acid- and N-methyl-D-aspartate-induced excitotoxicity in the retina of chicks. *J. Comp. Neurol.* 393, 1-15. 10.1002/(SICI)1096-9861(19980330)393:1<1::AID-CNE1>3.0.CO;2-3 9520096

[DEV202070C17] Fischer, A. J., Hendrickson, A. and Reh, T. A. (2001). Immunocytochemical characterization of cysts in the peripheral retina and pars plana of the adult primate. *Invest. Ophthalmol. Vis. Sci.* 42, 3256-3263. 11726631

[DEV202070C18] Fischer, A. J., Mcguire, C. R., Dierks, B. D. and Reh, T. A. (2002). Insulin and fibroblast growth factor 2 activate a neurogenic program in Müller glia of the chicken retina. *J. Neurosci.* 22, 9387-9398. 10.1523/JNEUROSCI.22-21-09387.2002 12417664 PMC6758014

[DEV202070C19] Fischer, A. J., Schmidt, M., Omar, G. and Reh, T. A. (2004). BMP4 and CNTF are neuroprotective and suppress damage-induced proliferation of Müller glia in the retina. *Mol. Cell. Neurosci.* 27, 531-542. 10.1016/j.mcn.2004.08.007 15555930

[DEV202070C20] Fischer, A. J., Scott, M. A., Ritchey, E. R. and Sherwood, P. (2009a). Mitogen-activated protein kinase-signaling regulates the ability of Müller glia to proliferate and protect retinal neurons against excitotoxicity. *Glia* 57, 1538-1552. 10.1002/glia.20868 19306360 PMC2775435

[DEV202070C21] Fischer, A. J., Scott, M. A. and Tuten, W. (2009b). Mitogen-activated protein kinase-signaling stimulates Müller glia to proliferate in acutely damaged chicken retina. *Glia* 57, 166-181. 10.1002/glia.20743 18709648 PMC2774719

[DEV202070C22] Fischer, A. J., Zelinka, C., Gallina, D., Scott, M. A. and Todd, L. (2014). Reactive microglia and macrophage facilitate the formation of Müller glia-derived retinal progenitors. *Glia* 62, 1608-1628. 10.1002/glia.22703 24916856 PMC4140984

[DEV202070C23] Fischer, A. J., Zelinka, C. and Milani-Nejad, N. (2015). Reactive retinal microglia, neuronal survival, and the formation of retinal folds and detachments. *Glia* 63, 313-327. 10.1002/glia.22752 25231952 PMC4268330

[DEV202070C24] Gallina, D., Todd, L. and Fischer, A. J. (2014). A comparative analysis of Müller glia-mediated regeneration in the vertebrate retina. *Exp. Eye Res.* 123, 121-130. 10.1016/j.exer.2013.06.019 23851023 PMC3887127

[DEV202070C25] Gallina, D., Palazzo, I., Steffenson, L., Todd, L. and Fischer, A. J. (2015). Wnt/β-catenin-signaling and the formation of Müller glia-derived progenitors in the chick retina. *Dev. Neurobiol.* 76, 983-1002. 10.1002/dneu.22370 26663639 PMC4900948

[DEV202070C71] Gallina, D., Palazzo, I., Steffenson, L., Todd, L. and Fischer, A. J. (2016). Wnt/β-catenin-signaling and the formation of Müller glia-derived progenitors in the chick retina. *Dev. Neurobiol.* 76, 983-1002. 10.1002/dneu.22370 26663639 PMC4900948

[DEV202070C26] Ge, S. X., Jung, D. and Yao, R. (2020). ShinyGO: a graphical gene-set enrichment tool for animals and plants. *Bioinformatics* 36, 2628-2629. 10.1093/bioinformatics/btz931 31882993 PMC7178415

[DEV202070C27] Ghai, K., Zelinka, C. and Fischer, A. J. (2009). Serotonin released from amacrine neurons is scavenged and degraded in bipolar neurons in the retina. *J. Neurochem.* 111, 1-14. 10.1111/j.1471-4159.2009.06270.x 19619137 PMC2774720

[DEV202070C28] Ghai, K., Zelinka, C. and Fischer, A. J. (2010). Notch signaling influences neuroprotective and proliferative properties of mature Müller glia. *J. Neurosci.* 30, 3101-3112. 10.1523/JNEUROSCI.4919-09.2010 20181607 PMC2834965

[DEV202070C29] Guca, E., Suñol, D., Ruiz, L., Konkol, A., Cordero, J., Torner, C., Aragon, E., Martin-Malpartida, P., Riera, A. and Macias, M. J. (2018). TGIF1 homeodomain interacts with Smad MH1 domain and represses TGF-β signaling. *Nucleic Acids Res.* 46, 9220-9235. 10.1093/nar/gky680 30060237 PMC6158717

[DEV202070C30] Hayes, S., Nelson, B. R., Buckingham, B. and Reh, T. A. (2007). Notch signaling regulates regeneration in the avian retina. *Dev. Biol.* 312, 300-311. 10.1016/j.ydbio.2007.09.046 18028900 PMC2170876

[DEV202070C31] Hitchcock, P. F. and Raymond, P. A. (1992). Retinal regeneration. *Trends Neurosci.* 15, 103-108. 10.1016/0166-2236(92)90020-9 1373917

[DEV202070C32] Hoang, T., Wang, J., Boyd, P., Wang, F., Santiago, C., Jiang, L., Yoo, S., Lahne, M., Todd, L. J., Jia, M. et al. (2020). Gene regulatory networks controlling vertebrate retinal regeneration. *Science* 370, eabb8598. 10.1126/science.abb8598 33004674 PMC7899183

[DEV202070C33] Huang, T., Cui, J., Li, L., Hitchcock, P. F. and Li, Y. (2012). The role of microglia in the neurogenesis of zebrafish retina. *Biochem. Biophys. Res. Commun.* 421, 214-220. 10.1016/j.bbrc.2012.03.139 22497888 PMC3354761

[DEV202070C34] Jorstad, N. L., Wilken, M. S., Grimes, W. N., Wohl, S. G., VandenBosch, L. S., Yoshimatsu, T., Wong, R. O., Rieke, F. and Reh, T. A. (2017). Stimulation of functional neuronal regeneration from Müller glia in adult mice. *Nature* 548, 103-107. 10.1038/nature23283 28746305 PMC5991837

[DEV202070C35] Jouannet, S., Saint-Pol, J., Fernandez, L., Nguyen, V., Charrin, S., Boucheix, C., Brou, C., Milhiet, P.-E. and Rubinstein, E. (2016). TspanC8 tetraspanins differentially regulate the cleavage of ADAM10 substrates, Notch activation and ADAM10 membrane compartmentalization. *Cell. Mol. Life Sci.* 73, 1895-1915. 10.1007/s00018-015-2111-z 26686862 PMC4819958

[DEV202070C36] Karl, M. O., Hayes, S., Nelson, B. R., Tan, K., Buckingham, B. and Reh, T. A. (2008). Stimulation of neural regeneration in the mouse retina. *Proc. Natl. Acad. Sci. USA* 105, 19508-19513. 10.1073/pnas.0807453105 19033471 PMC2614791

[DEV202070C37] Lebaron, R. G., Bezverkov, K. I., Zimber, M. P., Pavelec, R., Skonier, J. and Purchio, A. F. (1995). Beta IG-H3, a novel secretory protein inducible by transforming growth factor-β, is present in normal skin and promotes the adhesion and spreading of dermal fibroblasts in vitro. *J. Invest. Dermatol.* 104, 844-849. 10.1111/1523-1747.ep12607024 7738366

[DEV202070C38] Lemjabbar, H. and Basbaum, C. (2002). Platelet-activating factor receptor and ADAM10 mediate responses to Staphylococcus aureus in epithelial cells. *Nat. Med.* 8, 41-46. 10.1038/nm0102-41 11786905

[DEV202070C39] Ling, N., Ying, S.-Y., Ueno, N., Shimasaki, S., Esch, F., Hotta, M. and Guillemin, R. (1986). Pituitary FSH is released by a heterodimer of the β-subunits from the two forms of inhibin. *Nature* 321, 779-782. 10.1038/321779a0 3086749

[DEV202070C40] Mitra, S., Devi, S., Lee, M.-S., Jui, J., Sahu, A. and Goldman, D. (2022). Vegf signaling between Müller glia and vascular endothelial cells is regulated by immune cells and stimulates retina regeneration. *Proc. Natl. Acad. Sci. USA* 119, e2211690119. 10.1073/pnas.2211690119 36469778 PMC9897474

[DEV202070C41] Ooto, S., Akagi, T., Kageyama, R., Akita, J., Mandai, M., Honda, Y. and Takahashi, M. (2004). Potential for neural regeneration after neurotoxic injury in the adult mammalian retina. *Proc. Natl. Acad. Sci. USA* 101, 13654-13659. 10.1073/pnas.0402129101 15353594 PMC518808

[DEV202070C42] Osakada, F., Ooto, S., Akagi, T., Mandai, M., Akaike, A. and Takahashi, M. (2007). Wnt signaling promotes regeneration in the retina of adult mammals. *J. Neurosci.* 27, 4210-4219. 10.1523/JNEUROSCI.4193-06.2007 17428999 PMC6672527

[DEV202070C43] Palazzo, I., Deistler, K., Hoang, T. V., Blackshaw, S. and Fischer, A. J. (2020). NF-κB signaling regulates the formation of proliferating Müller glia-derived progenitor cells in the avian retina. *Development* 147, dev183418. 10.1242/dev.183418 32291273 PMC7325431

[DEV202070C44] Palazzo, I., Todd, L. J., Hoang, T. V., Reh, T. A., Blackshaw, S. and Fischer, A. J. (2022). NFkB-signaling promotes glial reactivity and suppresses Müller glia-mediated neuron regeneration in the mammalian retina. *Glia* 70, 1380-1401. 10.1002/glia.24181 35388544 PMC9585486

[DEV202070C45] Palazzo, I., Kelly, L., Koenig, L. and Fischer, A. J. (2023). Patterns of NFkB activation resulting from damage, reactive microglia, cytokines, and growth factors in the mouse retina. *Exp. Neurol.* 359, 114233. 10.1016/j.expneurol.2022.114233 36174748 PMC9722628

[DEV202070C46] Pollak, J., Wilken, M. S., Ueki, Y., Cox, K. E., Sullivan, J. M., Taylor, R. J., Levine, E. M. and Reh, T. A. (2013). Ascl1 reprograms mouse Müller glia into neurogenic retinal progenitors. *Development* 140, 2619-2631. 10.1242/dev.091355 23637330 PMC3666387

[DEV202070C47] Ramachandran, R., Zhao, X.-F. and Goldman, D. (2011). Ascl1a/Dkk/β-catenin signaling pathway is necessary and glycogen synthase kinase-3β inhibition is sufficient for zebrafish retina regeneration. *Proc. Natl. Acad. Sci. USA* 108, 15858-15863. 10.1073/pnas.1107220108 21911394 PMC3179085

[DEV202070C48] Raymond, P. A. (1991). Retinal regeneration in teleost fish. *Ciba Found. Symp.* 160, 171-186; discussion 186-91. 10.1002/9780470514122.ch9 1752162

[DEV202070C49] Reichenbach, A. and Bringmann, A. (2013). New functions of Müller cells. *Glia* 61, 651-678. 10.1002/glia.22477 23440929

[DEV202070C50] Ritchey, E. R., Bongini, R. E., Code, K. A., Zelinka, C., Petersen-Jones, S. and Fischer, A. J. (2010). The pattern of expression of guanine nucleotide-binding protein β3 in the retina is conserved across vertebrate species. *Neuroscience* 169, 1376-1391. 10.1016/j.neuroscience.2010.05.081 20538044 PMC2914127

[DEV202070C51] Satija, R., Farrell, J. A., Gennert, D., Schier, A. F. and Regev, A. (2015). Spatial reconstruction of single-cell gene expression data. *Nat. Biotechnol.* 33, 495-502. 10.1038/nbt.3192 25867923 PMC4430369

[DEV202070C52] Seegar, T. C. M., Killingsworth, L. B., Saha, N., Meyer, P. A., Patra, D., Zimmerman, B., Janes, P. W., Rubinstein, E., Nikolov, D. B., Skiniotis, G. et al. (2017). Structural basis for regulated proteolysis by the α-secretase ADAM10. *Cell* 171, 1638-1648.e7. 10.1016/j.cell.2017.11.014 29224781 PMC5773094

[DEV202070C53] Shannon, P., Markiel, A., Ozier, O., Baliga, N. S., Wang, J. T., Ramage, D., Amin, N., Schwikowski, B. and Ideker, T. (2003). Cytoscape: a software environment for integrated models of biomolecular interaction networks. *Genome Res.* 13, 2498-2504. 10.1101/gr.1239303 14597658 PMC403769

[DEV202070C54] Skonier, J., Bennett, K., Rothwell, V., Kosowski, S., Plowman, G., Wallace, P., Edelhoff, S., Disteche, C., Neubauer, M., Marquardt, H. et al. (1994). beta ig-h3: a transforming growth factor-β-responsive gene encoding a secreted protein that inhibits cell attachment in vitro and suppresses the growth of CHO cells in nude mice. *DNA Cell Biol.* 13, 571-584. 10.1089/dna.1994.13.571 8024701

[DEV202070C55] Stanke, J., Moose, H. E., El-Hodiri, H. M. and Fischer, A. J. (2010). Comparative study of Pax2 expression in glial cells in the retina and optic nerve of birds and mammals. *J. Comp. Neurol.* 518, 2316-2333. 10.1002/cne.22335 20437530 PMC3840394

[DEV202070C56] Thiel, W. A., Blume, Z. I. and Mitchell, D. M. (2022). Compensatory engulfment and Müller glia reactivity in the absence of microglia. *Glia* 70, 1402-1425. 10.1002/glia.24182 35451181 PMC9081278

[DEV202070C57] Todd, L. and Fischer, A. J. (2015). Hedgehog-signaling stimulates the formation of proliferating Müller glia-derived progenitor cells in the retina. *Development* 142, 2610-2622. 10.1242/dev.121616 26116667 PMC4529028

[DEV202070C58] Todd, L., Volkov, L. I., Zelinka, C., Squires, N. and Fischer, A. J. (2015). Heparin-binding EGF-like growth factor (HB-EGF) stimulates the proliferation of Müller glia-derived progenitor cells in avian and murine retinas. *Mol. Cell. Neurosci.* 69, 54-64. 10.1016/j.mcn.2015.10.004 26500021 PMC4658256

[DEV202070C59] Todd, L., Palazzo, I., Squires, N., Mendonca, N. and Fischer, A. J. (2017). BMP- and TGFβ-signaling regulate the formation of Müller glia-derived progenitor cells in the avian retina. *Glia* 65, 1640-1655. 10.1002/glia.23185 28703293 PMC5628513

[DEV202070C60] Todd, L., Suarez, L., Quinn, C. and Fischer, A. J. (2018). Retinoic acid-signaling regulates the proliferative and neurogenic capacity of Müller glia-derived progenitor cells in the avian retina. *Stem Cells* 36, 392-405. 10.1002/stem.2742 29193451 PMC5823757

[DEV202070C61] Todd, L., Palazzo, I., Suarez, L., Liu, X., Volkov, L., Hoang, T. V., Campbell, W. A., Blackshaw, S., Quan, N. and Fischer, A. J. (2019). Reactive microglia and IL1β/IL-1R1-signaling mediate neuroprotection in excitotoxin-damaged mouse retina. *J. Neuroinflammation* 16, 118. 10.1186/s12974-019-1505-5 31170999 PMC6555727

[DEV202070C62] Todd, L., Finkbeiner, C., Wong, C. K., Hooper, M. J. and Reh, T. A. (2020). Microglia suppress Ascl1-induced retinal regeneration in mice. *Cell Rep.* 33, 108507. 10.1016/j.celrep.2020.108507 33326790

[DEV202070C63] Ueki, Y., Wilken, M. S., Cox, K. E., Chipman, L., Jorstad, N., Sternhagen, K., Simic, M., Ullom, K., Nakafuku, M. and Reh, T. A. (2015). Transgenic expression of the proneural transcription factor Ascl1 in Müller glia stimulates retinal regeneration in young mice. *Proc. Natl. Acad. Sci. USA* 112, 13717-13722. 10.1073/pnas.1510595112 26483457 PMC4640735

[DEV202070C64] van Rooijen, N. (1992). Liposome-mediated elimination of macrophages. *Res. Immunol.* 143, 215-219. 10.1016/S0923-2494(92)80169-L 1533469

[DEV202070C65] Wan, J. and Goldman, D. (2016). Retina regeneration in zebrafish. *Curr. Opin. Genet. Dev.* 40, 41-47. 10.1016/j.gde.2016.05.009 27281280 PMC5135611

[DEV202070C66] Wan, J., Ramachandran, R. and Goldman, D. (2012). HB-EGF is necessary and sufficient for Müller glia dedifferentiation and retina regeneration. *Dev. Cell* 22, 334-347. 10.1016/j.devcel.2011.11.020 22340497 PMC3285435

[DEV202070C67] White, D. T., Sengupta, S., Saxena, M. T., Xu, Q., Hanes, J., Ding, D., Ji, H. and Mumm, J. S. (2017). Immunomodulation-accelerated neuronal regeneration following selective rod photoreceptor cell ablation in the zebrafish retina. *Proc. Natl. Acad. Sci. USA* 114, E3719-E3728. 10.1073/pnas.1617721114 28416692 PMC5422825

[DEV202070C68] Williams, S. A., Chen, L.-F., Kwon, H., Fenard, D., Bisgrove, D., Verdin, E. and Greene, W. C. (2004). Prostratin antagonizes HIV latency by activating NF-κB. *J. Biol. Chem.* 279, 42008-42017. 10.1074/jbc.M402124200 15284245

[DEV202070C69] Yao, K., Qiu, S., Tian, L., Snider, W. D., Flannery, J. G., Schaffer, D. V. and Chen, B. (2016). Wnt regulates proliferation and neurogenic potential of Müller glial cells via a Lin28/let-7 miRNA-dependent pathway in adult mammalian retinas. *Cell Rep.* 17, 165-178. 10.1016/j.celrep.2016.08.078 27681429 PMC5076887

[DEV202070C70] Zelinka, C. P., Scott, M. A., Volkov, L. and Fischer, A. J. (2012). The reactivity, distribution and abundance of non-astrocytic inner retinal glial (NIRG) cells are regulated by microglia, acute damage, and IGF1. *PLoS ONE* 7, e44477. 10.1371/journal.pone.0044477 22973454 PMC3433418

