# Supplementary material for: Heparin-binding epidermal growth factor and fibroblast growth factor 2 rescue Müller glia-derived progenitor cell formation in microglia- and macrophage-ablated chick retinas
Source: Development. 2023 Dec 6;150(23):dev202070. doi: 10.1242/dev.202070 (PMC10730090; doi:10.1242/dev.202070)
Supplement: Supplementary information [file develop-150-202070-s1.pdf]

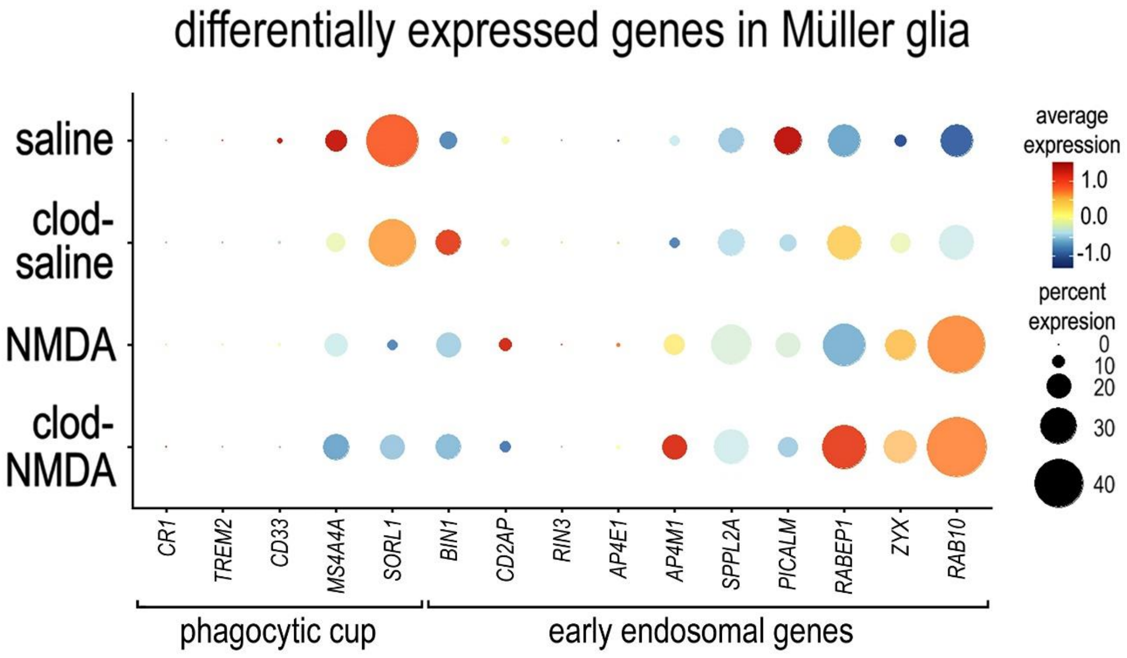

Fig. S1. Dot plot of genes related to phagocytic cups and early endosomal genes in MG in normal and damaged retinas with and without microglia. The dot plot illustrates expression levels (heatmap) and percent expressed (dot size) for different genes in MG that are significantly up- or downregulated. Significant of difference ($p<0.01$) was determined by using a Wilcox rank sum test with Bonferroni correction. *RABEP1* was the only gene that was significantly increased in MG in damaged retinas when microglia are absent. scRNA-seq was used to analyze patterns and levels of expression of retinoic acid- related genes in retinas treated with saline ± clodronate and NMDA ± clodronate.

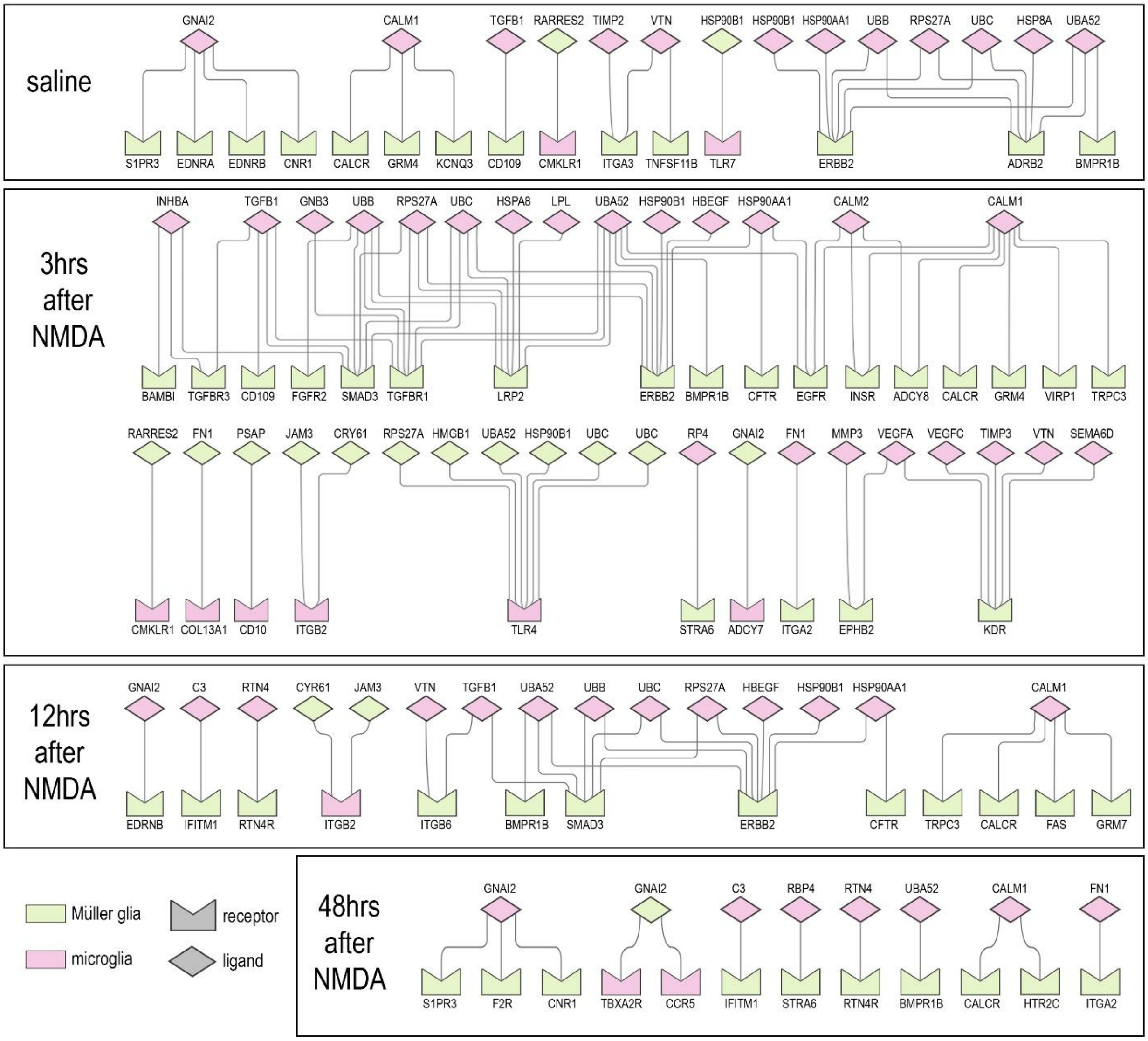

Fig. S2. Inferred autocrine ligand-receptor (LR) interactions between MG. scRNA-seq was used to identify putative LR interactions in control and NMDA-damaged retinas at 3, 12 and 48 hours after treatment. SingleCellSignalR was used to identify putative LR interactions. Significant LR-interactions between microglia and MG are illustrated for undamaged and damaged retinas with or without microglia.

Development • Supplementary information

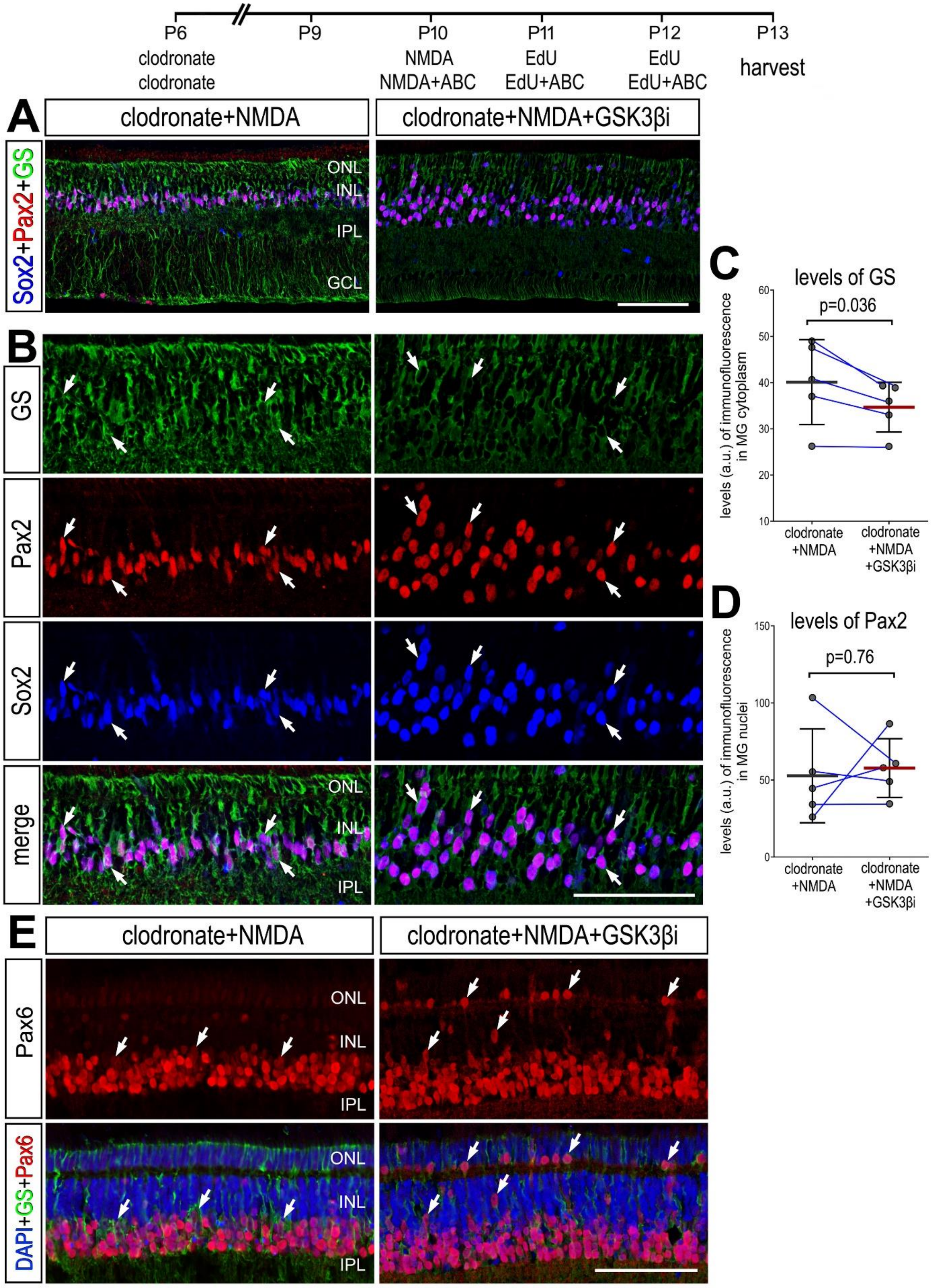
P6
clodronate
clodronate
P9
P10
NMDA
NMDA+ABC
P11
EdU
EdU+ABC
P12
EdU
EdU+ABC
P13
harvest
A
clodronate+NMDA
clodronate+NMDA+GSK3βi
Sox2+Pax2+GS
ONL
INL
IPL
GCL
B
GS
Pax2
Sox2
merge
C
levels of GS
p=0.036
levels (a.u.) of immunofluorescence in MG cytoplasm
clodronate +NMDA
clodronate +NMDA +GSK3βi
D
levels of Pax2
p=0.76
levels (a.u.) of immunofluorescence in MG nuclei
E
Pax6
DAPI+GS+Pax6

Fig. S3. Expression of MG markers in retinas missing microglia and treated with GSK3b inhibitors. We applied clodronate liposomes at P6, NMDA a cocktail of GSK3β inhibitors at P10, EdU GSK3β inhibitors at P11 and P12, and harvested retinas at P13. Retinal sections were labeled for glutamine synthetase (GS, green; **A,B,E**), Sox2 (blue; **A,B**), Pax2 (red; **A,B**), Pax6 (red; **E**) and DNA (DAPI, blue; **E**). The histograms in **C** and **D** illustrate the mean (bar ± SD) levels of fluorescence in the INL for GS and Pax2, each dot represents one biological replicate, and blue lines connect data points from control and treated retinas from the same individual. Significance of difference (p-values) was determined by using a paired t-test (**D**). Calibration bars in **A, B** and **E** represent 50 µm. Arrows indicate the nuclei of MG.

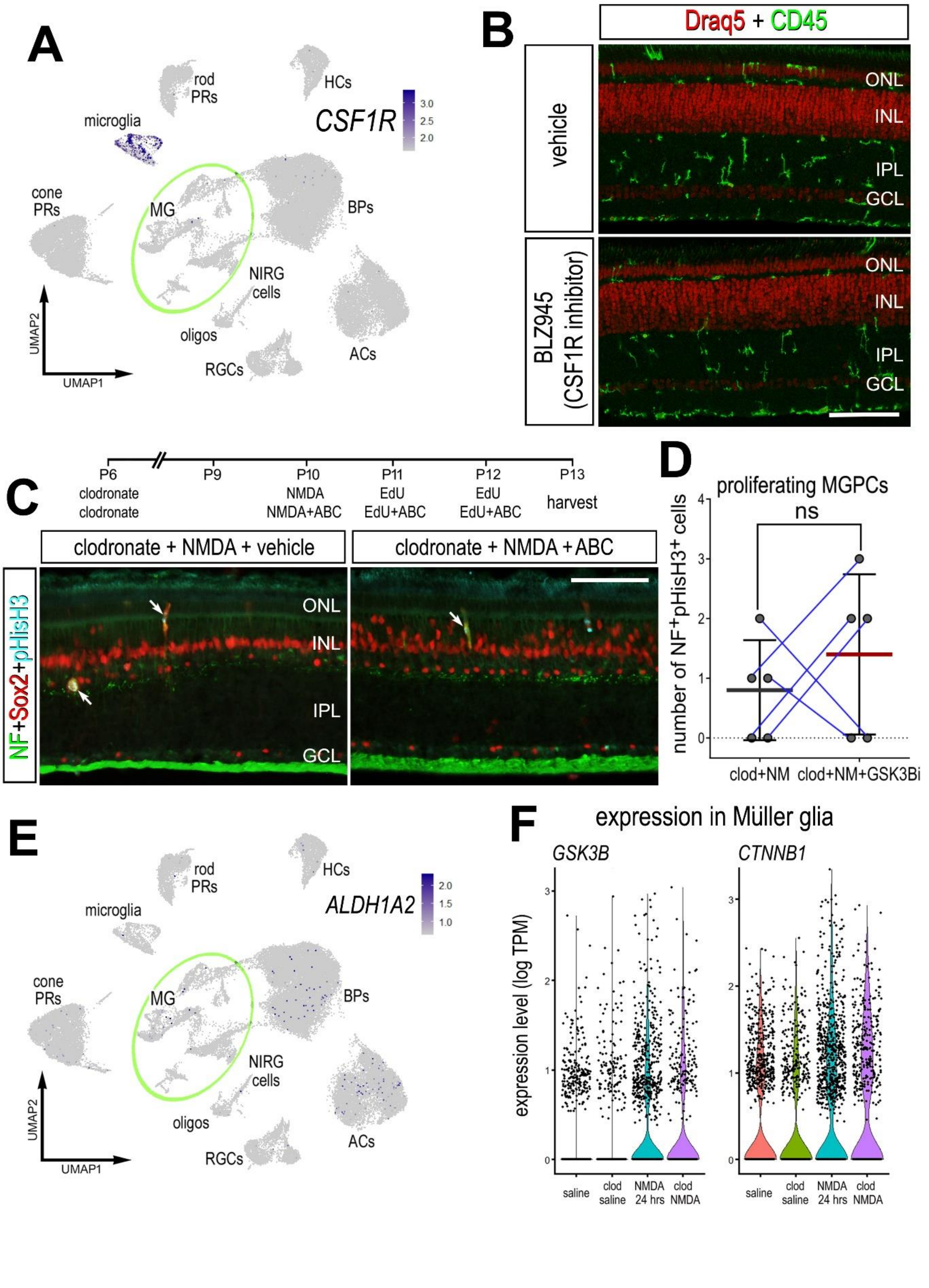
A
rod PRs
HCs
microglia
CSF1R
3.0
2.5
2.0
cone PRs
MG
BPs
NIRG cells
oligos
RGCs
ACs
UMAP2
UMAP1
B
Draq5 + CD45
vehicle
BLZ945 (CSF1R inhibitor)
ONL
INL
IPL
GCL
C
P6 clodronate clodronate
P9
P10 NMDA NMDA+ABC
P11 EdU EdU+ABC
P12 EdU EdU+ABC
P13 harvest
clodronate + NMDA + vehicle
clodronate + NMDA + ABC
NF+Sox2+pHisH3
D
proliferating MGPCs
ns
number of NF+pHisH3+ cells
clod+NM
clod+NM+GSK3Bi
E
ALDH1A2
2.0
1.5
1.0
F
expression in Müller glia
GSK3B
CTNNB1
expression level (log TPM)
saline
clod saline
NMDA 24 hrs
clod NMDA

Fig. S4. A: UMAP heatmap plot of *CSF1R* in chick retinal cells. See Figure 3A-D for legend. **B:** Three consecutive daily injections of BLZ945 (CSF1R inhibitor) has no effect on CD45-positive microglia in the chick retina. Retinal sections were labeled for DNA (Draq5; red) and microglia (CD45; green). **C:** GSK3B-inhibitors have no effect on the proliferation of MGPCs. Retinal sections were labeled for neurofilament (NF; green), Sox2 (red) and phospho-histone H3 (pHisH3; cyan). **D:** Histogram illustrating mean (±SD) numbers of proliferating MGPCs. Each dot represents one biological replicate and blue lines connect data points from control and treated retinas from the same individual. Significance of difference (p-value) was determined by using a paired t-test. **E:** UMAP heatmap plot of *ALDH1A2* in chick retinal cells. See Figure 3A-D for legend. **F:** Violin plots illustrating levels of expression of *GSK3B* and *CTNNB1* (β-catenin) in MG in undamaged retinas, undamaged retinas missing microglia, NMDA-damaged retinas, and NMDA-damaged retinas missing microglia. See figure 1C,D for legend.

Table S1. DEGs in MG in retinas treated with saline (pct.1) vs saline + clodronate-liposomes (pct.2). Negative avg log2FC represents an increase and positive avg log2FC represents a decrease.

Available for download at
https://journals.biologists.com/dev/article-lookup/doi/10.1242/dev.202070#supplementary-data

Table S2. DEGs in MG in retinas treated with NMDA (pct.1) vs NMDA + clodronate-liposomes (pct.2). Negative avg log2FC represents an increase and positive avg log2FC represents a decrease.

Available for download at
https://journals.biologists.com/dev/article-lookup/doi/10.1242/dev.202070#supplementary-data

Table S3. DEGs in MG in retinas treated with saline (pct.1) vs NMDA (pct.2). Negative avg log2FC represents an increase and positive avg log2FC represents a decrease.

Available for download at
https://journals.biologists.com/dev/article-lookup/doi/10.1242/dev.202070#supplementary-data

Table S4. DEGs in MG in retinas treated with saline or NMDA and retinas harvested at 3, 12 or 48 hours later. DEGs were identified for resting MG vs 3+12+48hr NMDA MG, resting MG vs 3hr NMDA MG, resting MG vs 12hr NMDA MG, and resting MG vs 48hr NMDA MG. Negative avg log2FC represents an increase and positive avg log2FC represents a decrease

Available for download at
https://journals.biologists.com/dev/article-lookup/doi/10.1242/dev.202070#supplementary-data

Table S5. Statistics for genes listed in Dot Plots in Figures 3, 4, 5, 6 and 7.

Figure 3f

```
FindMarkers(early_MG, ident.1 = "resting MG", ident.2 = c("NM3hr MG", "NM12hr MG", "NM48hr MG"), features = c("WNT4", "WNT5A", "WNT6", "SFRP1", "SFRP2", "WIF1", "FRZB", "FZD3", "CTNNB1"), min.pct = 0.01, logfc.threshold = 0.01)
```

	p_val	avg_log2FC	pct.1	pct.2	p_val_adj
FRZB	6.959940e-208	-0.73641069	0.018	0.458	1.297194e-203
WIF1	6.236237e-203	2.61261152	0.440	0.088	1.162310e-198
WNT6	1.352700e-162	-1.38506960	0.002	0.354	2.521163e-158
SFRP1	3.282211e-157	1.57848552	0.665	0.484	6.117386e-153
WNT4	5.402679e-94	-1.24108937	0.001	0.220	1.006951e-89
SFRP2	1.123826e-24	0.36664151	0.281	0.174	2.094588e-20
WNT5A	2.998765e-18	-0.03150130	0.064	0.154	5.589098e-14
CTNNB1	3.161820e-14	0.01736114	0.474	0.650	5.893001e-10
FZD3	1.251042e-12	-0.03469801	0.273	0.405	2.331692e-08

Figure 4a

```
FindMarkers(clod_MG, ident.1 = "NM", ident.2 = "clod_NM", features = c("FGF1", "FGF10", "FGF12", "FGFR1", "MAPK1", "MAPK6", "MAPKAPK2", "MAPKAPK3", "SPRY2"), group.by = "orig.ident", min.pct = 0.01, logfc.threshold = 0.01)
```

	p_val	avg_log2FC	pct.1	pct.2	p_val_adj
FGFR1	2.389008e-22	0.62776729	0.525	0.293	5.032684e-18
MAPK6	6.778693e-22	0.65951222	0.672	0.516	1.427999e-17
MAPKAPK3	1.328319e-14	-0.42574789	0.056	0.164	2.798237e-10
FGF1	9.925580e-09	-0.19130015	0.023	0.080	2.090923e-04
SPRY2	1.441824e-08	0.39691918	0.704	0.672	3.037347e-04
MAPKAPK2	2.496907e-07	0.29129051	0.392	0.265	5.259984e-03
FGF10	2.096280e-03	0.08484429	0.074	0.037	1.000000e+00
FGF12	9.736861e-02	-0.13235330	0.349	0.361	1.000000e+00
MAPK1	9.871330e-02	-0.08740614	0.352	0.369	1.000000e+00

```
FindMarkers(clod_MG, ident.1 = "SS", ident.2 = "clod_SS", features = c("FGF1", "FGF10", "FGF12", "FGFR1", "MAPK1", "MAPK6", "MAPKAPK2", "MAPKAPK3", "SPRY2"), group.by = "orig.ident", min.pct = 0.01, logfc.threshold = 0.01)
```

	p_val	avg_log2FC	pct.1	pct.2	p_val_adj
SPRY2	0.004937578	0.19666277	0.534	0.476	1
MAPK6	0.005254168	0.16366085	0.345	0.282	1
FGFR1	0.272221235	-0.09554117	0.457	0.473	1
MAPKAPK3	0.477902460	-0.03140568	0.135	0.146	1
MAPKAPK2	0.701824174	-0.02888859	0.187	0.177	1
MAPK1	0.712245015	-0.03744760	0.265	0.250	1
FGF12	0.783617334	-0.06499152	0.475	0.473	1
FGF1	0.909600032	-0.03651327	0.233	0.223	1

```
> FindMarkers(clod_MG, ident.1 = "SS", ident.2 = "NM", features = c("FGF1", "FGF10", "FGF12", "FGFR1", "MAPK1", "MAPK6", "MAPKAPK2", "MAPKAPK3", "SPRY2"), group.by = "orig.ident", min.pct = 0.01, logfc.threshold = 0.01)
```

	p_val	avg_log2FC	pct.1	pct.2	p_val_adj
MAPK6	1.268156e-76	-1.07038319	0.345	0.672	2.671497e-72
FGF1	3.654600e-55	0.71210447	0.233	0.023	7.698781e-51
SPRY2	2.862949e-41	-0.82939101	0.534	0.704	6.031087e-37
FGF12	1.402970e-24	0.60871379	0.475	0.349	2.955497e-20
MAPKAPK2	6.936962e-22	-0.34950784	0.187	0.392	1.461340e-17
FGF10	1.182789e-18	-0.15402384	0.003	0.074	2.491663e-14
MAPKAPK3	3.351993e-12	0.38807573	0.135	0.056	7.061308e-08
MAPK1	2.031448e-02	-0.04110462	0.265	0.352	1.000000e+00
FGFR1	3.621806e-01	0.03147393	0.457	0.525	1.000000e+00

Figure 4f

```
FindMarkers(early_MG, ident.1 = "resting MG", ident.2 = c("NM3hr MG", "NM12hr
MG", "NM48hr MG"), features = c("FGF1", "FGF10", "FGF12", "FGFR1", "MAPK1", "
MAPK6", "MAPKAPK2", "MAPKAPK3", "SPRY2"), min.pct = 0.01, logfc.threshold = 0
.01)
                  p_val  avg_log2FC pct.1 pct.2     p_val_adj
MAPK6    2.102593e-201 -0.94257615 0.720 0.907 3.918812e-197
FGF1     3.749518e-125  0.66710640 0.345 0.083 6.988351e-121
SPRY2     1.651956e-78 -0.53785944 0.378 0.668  3.078915e-74
FGF10     4.232294e-65 -0.22456243 0.013 0.186  7.888149e-61
FGF12     1.192070e-22  0.58884530 0.531 0.514  2.221780e-18
MAPKAPK2  2.459611e-20 -0.04393262 0.323 0.494  4.584222e-16
MAPK1     2.625855e-12  0.05418908 0.291 0.432  4.894068e-08
MAPKAPK3  3.676987e-07  0.17989025 0.229 0.186  6.853168e-03
FGFR1     7.442556e-05  0.11258808 0.464 0.590  1.000000e+00

FindMarkers(early_MG, ident.1 = "resting MG", ident.2 = c("NM3hr MG"), featur
es = c("FGFR1"), min.pct = 0.01, logfc.threshold = 0.01)
              p_val avg_log2FC pct.1 pct.2    p_val_adj
FGFR1 1.571866e-68 -0.5205046 0.464 0.895 2.929645e-64

> FindMarkers(early_MG, ident.1 = "NM3hr MG", ident.2 = c("NM12hr MG"), featu
res = c("FGFR1"), min.pct = 0.01, logfc.threshold = 0.01)
               p_val avg_log2FC pct.1 pct.2     p_val_adj
FGFR1 2.705122e-143   1.056656 0.895 0.362 5.041806e-139
```

Figure 5a

```
FindMarkers(clod_MG, ident.1 = "NM", ident.2 = "clod_NM", features = c("HBEGF
", "EGFR", "ERBB2", "GRB2", "ADAM9", "ADAM10"), group.by = "orig.ident", min.
pct = 0.01, logfc.threshold = 0.01)

              p_val  avg_log2FC pct.1 pct.2    p_val_adj
HBEGF  1.625671e-18  0.77767332 0.513 0.315 3.424639e-14
ADAM9  4.320135e-06 -0.29391801 0.148 0.230 2.100796e-02
ERBB2  1.052811e-01 -0.02559355 0.085 0.061 1.000000e+00
GRB2   1.366727e-01  0.08106176 0.427 0.376 1.000000e+00
EGFR   1.480282e-01  0.16956444 0.070 0.052 1.000000e+00
ADAM10 9.657403e-01  0.01588233 0.354 0.340 1.000000e+00

FindMarkers(clod_MG, ident.1 = "SS", ident.2 = "NM", features = c("HBEGF", "E
GFR", "ERBB2", "GRB2", "ADAM9", "ADAM10"), group.by = "orig.ident", min.pct =
0.01, logfc.threshold = 0.01)

               p_val  avg_log2FC pct.1 pct.2     p_val_adj
HBEGF  3.164245e-147 -1.75104411 0.029 0.513 6.665799e-143
ADAM10  1.293643e-14 -0.34865857 0.185 0.354  2.725188e-10
EGFR    3.166742e-06 -0.26694283 0.028 0.070  6.671059e-02
ERBB2   9.956314e-05 -0.04553082 0.043 0.085  1.000000e+00
ADAM9   3.187400e-01  0.11112578 0.152 0.148  1.000000e+00
GRB2    4.568791e-01  0.06004539 0.356 0.427  1.000000e+00

FindMarkers(clod_MG, ident.1 = "SS", ident.2 = "clod_SS", features = c("HBEGF
", "EGFR", "ERBB2", "GRB2", "ADAM9", "ADAM10"), group.by = "orig.ident", min.
pct = 0.01, logfc.threshold = 0.01)

              p_val  avg_log2FC pct.1 pct.2   p_val_adj
EGFR   2.034004e-07 -0.37998802 0.028 0.079 0.052848321
HBEGF  4.074582e-02 -0.10596081 0.029 0.046 1.000000000
ADAM10 8.950877e-02  0.08350485 0.185 0.154 1.000000000
GRB2   3.892708e-01  0.05382874 0.356 0.329 1.000000000
ADAM9  6.373910e-01 -0.04745526 0.152 0.142 1.000000000
```

Development • Supplementary information

```
ERBB2  7.330943e-01  0.01017778 0.043 0.040 1.000000000
```

Figure 5f

```
FindMarkers(early_MG, ident.1 = "resting MG", ident.2 = c("NM3hr MG", "NM12hr MG", "NM48hr MG"), features = c("HBEGF", "EGFR", "ERBB2", "GRB2", "ADAM9", "ADAM10"), min.pct = 0.01, logfc.threshold = 0.01)

              p_val   avg_log2FC pct.1 pct.2    p_val_adj
HBEGF  0.000000e+00 -2.43455330 0.105 0.800 0.000000e+00
ADAM10 4.071952e-58 -0.20833239 0.396 0.667 7.589304e-54
GRB2   5.707613e-29 -0.14596770 0.416 0.625 1.063785e-24
ERBB2  2.394689e-13 -0.06026036 0.095 0.179 4.463222e-09
ADAM9  1.967558e-07  0.11057057 0.336 0.460 3.667135e-03
EGFR   2.557701e-03  0.15756717 0.101 0.137 1.000000e+00
```

Figure 6a

```
FindMarkers(clod_MG, ident.1 = "NM", ident.2 = "clod_NM", features = c("TGFB1", "TGFB2", "TGFB3", "TGIF1", "TGFBI", "INHBA", "TGFBR1", "TGFBR3"), group.by = "orig.ident", min.pct = 0.01, logfc.threshold = 0.01)

              p_val   avg_log2FC pct.1 pct.2    p_val_adj
INHBA  2.364005e-48  1.77369554 0.538 0.195 4.980014e-44
TGIF1  3.698492e-08  0.20758246 0.151 0.059 7.791242e-04
TGFBI  3.136353e-03  0.21273167 0.265 0.199 1.000000e+00
TGFB3  8.859048e-03  0.09388296 0.202 0.143 1.000000e+00
TGFBR3 1.194288e-02  0.09547510 0.104 0.066 1.000000e+00
TGFB1  2.484112e-01 -0.07615353 0.215 0.233 1.000000e+00
TGFBR1 3.818484e-01 -0.10358408 0.120 0.132 1.000000e+00
```

```
FindMarkers(clod_MG, ident.1 = "SS", ident.2 = "NM", features = c("TGFB1", "TGFB2", "TGFB3", "TGIF1", "TGFBI", "INHBA", "TGFBR1", "TGFBR3"), group.by = "orig.ident", min.pct = 0.01, logfc.threshold = 0.01)

               p_val   avg_log2FC pct.1 pct.2     p_val_adj
TGFB2  5.993809e-184 -2.74140693 0.010 0.567 1.262656e-179
INHBA   6.053987e-41 -1.12075888 0.268 0.538  1.275333e-36
TGFB1   1.466371e-33 -0.50690521 0.042 0.215  3.089057e-29
TGIF1   8.431249e-14 -0.17450503 0.052 0.151  1.776127e-09
TGFBI   2.784096e-08 -0.21372639 0.155 0.265  5.864978e-04
TGFB3   9.918568e-05 -0.07982203 0.129 0.202  1.000000e+00
TGFBR1  1.010689e-04 -0.06689487 0.068 0.120  1.000000e+00
TGFBR3  7.099843e-03 -0.02935907 0.069 0.104  1.000000e+00
```

```
FindMarkers(clod_MG, ident.1 = "SS", ident.2 = "clod_SS", features = c("TGFB1", "TGFB2", "TGFB3", "TGIF1", "TGFBI", "INHBA", "TGFBR1", "TGFBR3"), group.by = "orig.ident", min.pct = 0.01, logfc.threshold = 0.01)

              p_val   avg_log2FC pct.1 pct.2    p_val_adj
INHBA  1.690472e-18 -0.65211747 0.268 0.453 3.561148e-14
TGFB3  3.061774e-13 -0.55909175 0.129 0.254 6.449933e-09
TGFB1  9.295067e-04  0.25539001 0.042 0.015 1.000000e+00
TGFBI  1.365135e-02 -0.18029643 0.155 0.196 1.000000e+00
TGFBR1 1.728320e-02 -0.14291746 0.068 0.097 1.000000e+00
TGFBR3 4.559136e-02 -0.11441153 0.069 0.092 1.000000e+00
TGFB2  3.887020e-01 -0.02322199 0.010 0.014 1.000000e+00
TGIF1  4.802866e-01  0.02120620 0.052 0.060 1.000000e+00
```

Figure 6f

```
> FindMarkers(early_MG, ident.1 = "resting MG", ident.2 = c("NM3hr MG", "NM12hr MG", "NM48hr MG"), features = c("TGFB1", "TGFB2", "TGFB3", "TGIF1", "TGFBI", "INHBA", "TGFBR1", "TGFBR3"), min.pct = 0.01, logfc.threshold = 0.01)
```

Development • Supplementary information

```
              p_val  avg_log2FC pct.1 pct.2    p_val_adj
TGFB2  0.000000e+00 -2.80796976 0.070 0.664 0.000000e+00
TGFB1  3.722150e-75 -0.53946239 0.055 0.275 6.937344e-71
TGIF1  9.918582e-36 -0.17418847 0.159 0.339 1.848625e-31
TGFBI  3.219732e-21 -0.15514364 0.263 0.416 6.000936e-17
INHBA  1.677756e-19 -0.42693991 0.360 0.509 3.127001e-15
TGFBR1 2.424136e-17 -0.02006268 0.104 0.210 4.518105e-13
TGFBR3 2.854405e-02  0.09385214 0.061 0.081 1.000000e+00
```

Figure 7b

```
FindMarkers(clod_MG, ident.1 = "NM", ident.2 = "clod_NM", features = c("RARA"
, "RARB", "RXRA", "RXRG", "CYP26A1", "ALDH1A1"), group.by = "orig.ident", min
.pct = 0.01, logfc.threshold = 0.01)
```

```
               p_val  avg_log2FC pct.1 pct.2    p_val_adj
ALDH1A1 4.014712e-09 -0.15988594 0.005 0.044 8.457392e-05
RARA    2.342166e-07  0.12312201 0.064 0.009 4.934007e-03
RXRG    4.280897e-04  0.15995838 0.308 0.221 1.000000e+00
CYP26A1 2.914654e-02 -0.02588425 0.025 0.044 1.000000e+00
RXRA    4.429671e-02 -0.15505815 0.247 0.279 1.000000e+00
RARB    2.654839e-01 -0.16330687 0.392 0.389 1.000000e+00
```

```
FindMarkers(clod_MG, ident.1 = "SS", ident.2 = "clod_SS", features = c("RARA"
, "RARB", "RXRA", "RXRG", "CYP26A1", "ALDH1A1"), group.by = "orig.ident", min
.pct = 0.01, logfc.threshold = 0.01)
```

```
               p_val  avg_log2FC pct.1 pct.2    p_val_adj
ALDH1A1 1.605357e-12  0.28585516 0.077 0.006 3.381845e-08
CYP26A1 4.308991e-08  0.37365838 0.115 0.044 9.077320e-04
RXRA    8.977831e-07 -0.41052555 0.177 0.262 1.891270e-02
RARB    1.764386e-05 -0.30634557 0.511 0.586 3.716855e-02
RXRG    3.095736e-02 -0.19976282 0.243 0.273 1.000000e+00
RARA    3.418922e-01  0.01756251 0.026 0.019 1.000000e+00
```

```
FindMarkers(clod_MG, ident.1 = "SS", ident.2 = "NM", features = c("RARA", "RA
RB", "RXRA", "RXRG", "CYP26A1", "ALDH1A1"), group.by = "orig.ident", min.pct
= 0.01, logfc.threshold = 0.01)
```

```
               p_val  avg_log2FC pct.1 pct.2    p_val_adj
RARB    1.054873e-20  0.58908201 0.511 0.392 2.222196e-16
ALDH1A1 2.519863e-19  0.31430641 0.077 0.005 5.308344e-15
CYP26A1 8.144630e-19  0.51024058 0.115 0.025 1.715748e-14
RARA    2.152713e-05 -0.05280525 0.026 0.064 4.534904e-01
RXRA    3.245594e-03 -0.09170134 0.177 0.247 1.000000e+00
RXRG    6.282007e-02  0.02975153 0.243 0.308 1.000000e+00
```

Figure 7f

```
FindMarkers(early_MG, ident.1 = "resting MG", ident.2 = c("NM3hr MG", "NM12hr
MG", "NM48hr MG"), features = c("RARA", "RARB", "RXRA", "RXRG", "CYP26A1", "A
LDH1A1"), min.pct = 0.01, logfc.threshold = 0.01)
```

```
                p_val avg_log2FC pct.1 pct.2     p_val_adj
ALDH1A1 1.982134e-123 0.62394228 0.248 0.029 3.694301e-119
RARB     1.449676e-42 0.51080248 0.328 0.184  2.701906e-38
RARA     7.375380e-11 0.36766529 0.234 0.262  2.308104e-06
CYP26A1  6.445438e-10 0.09417273 0.048 0.017  1.201301e-05
RXRG     2.026445e-07 0.28632706 0.331 0.294  3.776889e-03
RXRA     7.705532e-02 0.11932525 0.152 0.182  1.000000e+00
```

Table S6. Lists of significant autocrine LR-interactions in MG and Venn diagram lists for figure 2.

Available for download at
https://journals.biologists.com/dev/article-lookup/doi/10.1242/dev.202070#supplementary-data

Table S7. Antibodies, working dilution, clone/catalog number and source.

Antibody	Dilution	Host	Clone/Catalog number	Source
Sox2	1:1000	Goat	KOY0418121	R&D
Sox9	1:2000	Rabbit	AB5535	Millipore
Pax6	1:50	Mouse	PAX6	DSHB
Pax2	1:50	Rabbit	AB5535	Millipore
Nuclear beta catenin	1:40	Mouse	PY654	DSHB
CD45	1:200	Mouse	HIS-C7	Prionics
Glutamine synthetase	1:1000	Mouse	610517	BD Biosciences
phospho-histone H3	1:600	Rabbit	06-570	Millipore
Anti-goat IgG Alexa Fluor 488	1:1000	Donkey	A3214	Life Technologies
Anti-goat IgG Alexa Fluor 568	1:1000	Donkey	A-11057	Life Technologies
Anti-rabbit IgG Alexa Fluor 488	1:1000	Goat	A32731	Life Technologies
Anti-rabbit IgG Alexa Fluor 568	1:1000	Goat	A-11036	Life Technologies
Anti-rabbit IgG Alexa Fluor 647	1:1000	Goat	A32733	Life Technologies
Anti-mouse IgG Alexa Fluor 488	1:1000	Goat	A32723	Life Technologies
Anti-mouse IgG Alexa Fluor 568	1:1000	Goat	A-11004	Life Technologies
Anti-mouse IgG Alexa Fluor 647	1:1000	Goat	A32728	Life Technologies

All primary antibodies provided reproducible patterns of labeling, match patterns of expression in scRNA-seq libraries, and have been validated elsewhere
(Fischer et al., 2009a; Fischer et al., 2009b; Fischer et al., 2014; Gallina et al., 2015; Stanke et al., 2010).

Development • Supplementary information
