# Supplementary material for: Heparin-binding epidermal growth factor and fibroblast growth factor 2 rescue Müller glia-derived progenitor cell formation in microglia- and macrophage-ablated chick retinas
Source: Development. 2023 Dec 6;150(23):dev202070. doi: 10.1242/dev.202070 (PMC10730090; doi:10.1242/dev.202070)
Supplement: Supplementary file 1 [file develop-150-202070_review_history.pdf]

Heparin-binding epidermal growth factor and fibroblast growth factor 2 rescue Müller glia-derived progenitor cell formation in microglia- and macrophage-ablated chick retinas

Heithem El-Hodiri, James R Bentley, Alana G Reske, Olivia B Taylor, Isabella Palazzo, Warren A Campbell, Nicklaus R Halloy and Andy J Fischer
DOI: 10.1242/dev.202070

Editor: Francois Guillemot

Review timeline

Original submission: 6 July 2023
Editorial decision: 17 August 2023
First revision received: 18 September 2023
Editorial decision: 1 November 2023
Second revision received: 1 November 2023
Accepted: 2 November 2023

Original submission

First decision letter

MS ID#: DEVELOP/2023/202070

MS TITLE: Formation of Muller glia-derived progenitor cells in retinas depleted of microglia

AUTHORS: Heithem El-Hodiri, James R Bentley, Alana G Reske, Isabella Palazzo, Warren A Campbell, Nicklaus R Halloy, and Andy J Fischer

I have now received the reports of three referees on your manuscript and I have reached a decision. The referees' comments are appended below, or you can access them online: please go to BenchPress and click on the 'Manuscripts with Decisions' queue in the Author Area.

As you will see, all the referees express great interest in your work, but they also have significant criticisms and recommend a substantial revision of your manuscript before we can consider publication. If you are able to revise the manuscript along the lines suggested, which may involve further experiments, I will be happy to receive a revised version of the manuscript. Your revised paper will be re-reviewed by the original referees, and its acceptance will depend on your addressing satisfactorily all their major concerns. Please also note that Development will normally permit only one round of major revision.

If it would be helpful, you are welcome to contact us to discuss your revision in greater detail. Please send us a point-by-point response indicating your plans for addressing the referees' comments, and we will look over this and provide further guidance.

Please attend to all of the reviewers' comments and ensure that you clearly highlight all changes made in the revised manuscript. Please avoid using 'Tracked changes' in Word files as these are lost in PDF conversion. I should be grateful if you would also provide a point-by-point response detailing how you have dealt with the points raised by the reviewers in the 'Response to Reviewers' box. If you do not agree with any of their criticisms or suggestions please explain clearly why this is so.

© 2023. Published by The Company of Biologists under the terms of the Creative Commons Attribution License (https://creativecommons.org/licenses/by/4.0/).

Reviewer 1

Advance summary and potential significance to field

Previous studies by the Fischer lab have shown retinal injury in the postnatal chick stimulates MG delamination and proliferation. They also provided evidence that microglia are necessary for this proliferative response, and this could be overcome by activating Nfkb signaling in MG. They also previously performed scRNAseq on retinas with and without microglia ablation and suggested it impacted NFkb signaling in MG. This group showed Hbegf stimulates MG reprogramming, while insulin and Fgf2 can stimulate MG proliferation in the uninjured chick retina. They also previously developed a chlodronate-based method for microglia ablation that is very effective.
In the present study by El-Hodiri et al., microglia-dependent regulation of MG gene expression and proliferation in uninjured and injured chick retina was investigated. They ablate microglia with clodronate-liposomes. scRNAseq data was then used to identify gene expression changes in microglia, MG, and MG progenitors. Bioinformatic analysis of scRNAseq data sets not only identified microglia-dependent gene expression changes in MG, but also allowed for bioinformatic prediction of ligand receptor interactions. The main finding presented here is that inhibition of GSK3b, which stimulates MG proliferation in the injured retina, only stimulates MG delamination in the microglia-depleted and injured retina, while intravitreal injection of Fgf2 or Hbegf, stimulates delamination and proliferation of MG in the microglia-depleted retina. Further Smad3 inhibition and activation of RAR signaling resulted in a partial rescue of MG proliferation in the microglia depleted retina. Although these are interesting observations, they remain descriptive and the mechanisms underlying their action remain uncharacterized.

Comments for the author

In addition to the descriptive nature of this manuscript, the following concerns are noted.
Major concerns
1. Results: Lines 225-246 have a lot of redundancy and should be rewritten for conciseness and clarity.
2. Relying on clodronate liposomes as the only method to remove microglia is worrisome as it stimulates death of phagocytic cells not just microglia. There is a report indicating that removal of retinal microglia enhances the phagocytic activity of Muller glia (Glia 70:1402, 2022). Additional approaches to ablating retinal microglia are important to convincingly demonstrate the results obtained are due to microglia ablation and not from off-target effects on MG or other phagocytic cell types. Most studies employ PLX3395 or some more recent version of this drug that blocks the csf1r receptor and it would seem this should be applied to this study to ensure off-target effects of clodronate don't underlie some of their results.
3. Fig. 2 suggests tgfb-smad3 signaling takes place between microglia and MG in the injured retina; however, in the chlodrinate treated retinas that lack microglia, they employ Tgfb/Smad inhibitor (SIS3) to see if they can rescue the proliferation defect resulting from microglia ablation, but these chlodrinate treated retinas don't have microglia. This experiment does not make sense.
4. What receptors mediate the action of Fgf2 and Hbegf in the chick retina?
Please show ligand and receptor gene expression from the scRNAseq data so one can determine the cell types and level of expression of putative ligand receptor pairs. Since they both act to stimulate MG progenitor formation in absence of microglia, do they converge on the same signaling cascade? It seems the data is suggesting these ligands activate receptors on MG that are normally engaged by factors released from microglia. What microglia candidates fit these criteria and what happens when they are overexpressed or introduced (recombinant protein) in the chick retina? Also, why are Fgf ligands downregulated when it seems the authors are proposing Fgf signaling is critical to MG proliferation. What is the endogenous ligand if Fgf2 is not normally expressed in the chick retina?
5. Does apoptosis correlate with the different treatments affecting MG proliferation in injured and microglia-depleted retinas?
6. Wnt4 and 6 are the most highly down-regulated genes when microglia are ablated. Does this correlate with changes in MG nuclear b-catenin?
7. Does microglia ablation result in changes in b-catenin nuclear localization in the injured retina? Also, it would be nice to show the impact Gsk3b inhibition has on b-catenin nuclear localization.

© 2023. Published by The Company of Biologists under the terms of the Creative Commons Attribution License (https://creativecommons.org/licenses/by/4.0/).

8. Previous studies suggested a role for NFkb signaling in mediating the effects of microglia and MG. How does Fgf2 and Hbegf affect Nfkb signaling in MG? Are they acting upstream or downstream of Nfkb signaling? What happens if add Nfkb inhibitors and treat retinas with either Fgf2 or Hbegf in microglia-depleted injured retinas?
9. The title is misleading and implies MG-derived progenitors are formed independent of microglia; rather, the paper indicates microglia are necessary for MG progenitor formation and that if microglia are depleted this can be rescued by Fgf2 or Hbegf treatments. Maybe something like: Fgf2 and Hbegf rescue MG progenitor formation in the microglia-depleted chick retina.
Minor comments:
1. Line 254: indicates upregulation of Notch signaling genes in MG with microglia ablation, yet abstract states that these genes are upregulated in MG from microglia ablated resting retina. This is confusing.
2. 1st result section text is extremely repetitive. Everything has been said twice.
3. Line 278…incomplete sentence?
4. Line 332, MGPC1 should be MGPC2.
5. Line 334, SRFP should be SFRP.
6. Line 436, without retina should be without microglia.
7. Lines 362-369: "…FGF/MAPK-signaling were upregulated by MG in damaged retinas and this upregulation is diminished when microglia are absent (Fig. 1a)………..." I don't see this data in Fig 1a. Do you mean Fig. 4a? also correct the remaining figure refs in this paragraph.
8. Line 527, "mt" should be "must"
9. Please include microglia ablation protocol in methods.
10. Why are there no microglia in the UMAP plots for Fig. 1a, b?
11. Check entry error in table 2, lines 396 and 979; probably good idea to check all the entries.
12. Fig. 2e, f: what is the significance of LR pairs shown next to the pie charts?
13. Fig. 3k: what is "ABC"? I think it is the cocktail of inhibitors (GSK3b) but it would be nice to define in the figure legend and text.
14. In the discussion (line 549) they say proliferation is always associated with nuclear migration, but both with Smad3 inhibition and RARa activation, they see a small but significant increase in proliferation which is not coupled with delamination.

<u>Reviewer 2</u>

Advance summary and potential significance to field

This study by El-Hodiri and colleagues provides a potentially valuable addition to our understanding of how microglia/macrophages contribute to formation of Muller glia-derived progenitor cells in damaged chick retina by use of scRNAseq to analyze gene expression changes in Muller glia after depletion of microglia/macrophages both with and without damage. The focus on analysis of signaling pathways is relevant and key signals are tested for their ability to rescue the effects of microglia/macrophage depletion.

Comments for the author

While the study is interesting with some novel findings, the writing is often unclear, and the sources of data is some cases are not described. Some errors in the writing are noted here, but the manuscript would benefit from careful review. More detail about experimental design, especially the experiments involving scRNA-seq analysis is needed in the methods. Revisions, largely to the writing, would add impact to the study.

1. Better clarity around the use of the term microglia versus microglia/macrophages is needed. This is not clarified until the beginning of the results where it is stated that the term "microglia" will refer to both. It should be more clear in the title, abstract and introduction that both populations are potentially depleted/involved. Also, in the methods it is not clear if the single cell markers listed for microglia are also interpreted as applying to macrophages. Given the growing appreciation for the distinction between these cell populations it is important to be accurate in how they are referred to. It is pretty squishy throughout the paper which is problematic.

2. In the abstract, a bit more context in a few spots would help the reader. For example, since the study is in chick, after the first sentence it would be helpful to state something like: "In

© 2023. Published by The Company of Biologists under the terms of the Creative Commons Attribution License (https://creativecommons.org/licenses/by/4.0/).

chick, depletion of microglia prevents formation of proliferating MGPCs in response to damage." Also, stating at the outset that the analysis focused on changes in signaling pathways would give more focus to the results and conclusions.

3. Figure 1 – are microglia/macrophages identified in the scRNAseq data? One would expect that they would be present in the saline samples. They are not identified in the figure and not mentioned. Is there evidence of depletion from the data? As noted above, markers for microglia are mentioned in the methods section describing analysis of single cell data. Since depletion precedes damage, is there evidence for infiltration of macrophages in response to damage?

4. Line 269-271 is confusing: "Numbers of LR- interactions (significant upregulation of putative ligand and receptor) between cell types in the different treatment groups varied between 70 and 315." Looking at the figure it is not clear where these numbers come from.

5. Line 276: Fig. 2a,b,f should instead read: Fig. 2a,b,e. In general, the description of Figure 2a-f (lines 273-280) is confusing and should be clarified and tightened up. For example, this is not a sentence: "When microglia are ablated (Fig. 2a, e)." And the description jumps from resting to activated then apparently back to resting MG again. It is difficult to follow the signaling pathways being described on the chord diagrams, and the description didn't always seem to correspond to the figure. This section needs improvement, and the differences between the conditions in the chord diagrams could be indicated in some way.

6. Figure 2g described analysis of scRNAseq data at 3, 12 and 48 hrs after damage. Was this experiment done for this study? It is not described anywhere in the methods and data are not shown in the results before mention in this panel. The legend for Figure 2g states: "Representative significant LR-interactions between microglia and MG are illustrated for undamaged and damaged retinas with or without microglia." This seems inaccurate. There can be no interactions between microglia and MG without microglia.

7. Lines 295-296: "Wnt-ligands, WNT4 and WNT6, are among the most significantly downregulated genes in MG in damaged retinas where microglia were ablated." Are there data shown to support this statement? No figure, panel or table are referred to. This seems to be the rationale for the pivot to the analysis of Wnt pathways in Figure 3. .

8. Lines 324-325 state: "…we a probed large aggregate scRNA-seq library of MG (>70,000 cells) with glia bioinformatically isolated from retinas…." The source of these data are entirely unclear. Does this represent yet another scRNA-seq experiment? With enough sequencing to yield 70,000 MG for analysis? That is how this reads. None of this is described in the results or methods. If this is from previously published data there is no citation or GEO dataset identified.

9. The title may not best capture the focus of this study. The analysis alternates between effects on MG of microglia/macrophage depletion, and also effects on retinal gene expression and MG at different times after damage (eg. Figures 3-6). This actually makes it difficult to read since the rationale for the transitions are not always well explained. The more unifying theme is the focus on analysis of signaling pathways under these conditions.

10. Lines 435-436: "We probed for changes in expression levels of TGFB-related genes in MG in normal and damaged retinas, with and without retinas". Correction needed.

11. Primary single cell RNA-seq data files should be submitted to NCBI GEO.

Reviewer 3

Advance summary and potential significance to field

El-Hodiri et. al. probe regulatory mechanisms of pathways linked to Müller glia (MG) activation following injury that are contingent on microglia presence. This is an incompletely resolved question of high importance to the field of retinal regeneration, as microglia have been documented to exert species-specific and context-specific influences on the outcome of MG

© 2023. Published by The Company of Biologists under the terms of the Creative Commons Attribution License (https://creativecommons.org/licenses/by/4.0/).

reprogramming. A greater understanding of these pathways would prove meaningful to efforts to expand the regenerative competence of mammalian MG.

The authors rely on the chick model of NMDA-induced injury, for which they have characterized microglia-MG interactions to some extent in previous publications.
The interrogated pathways largely stem from candidate regulators identified across numerous scRNA-seq datasets. The work presented is significant for the field of retina regeneration as the authors detail a function (or lack thereof) for many of the identified pathways in driving MG proliferation and/or migration in the presence/absence of microglia. These include a series of agonist-driven experiments (WNT, FGF, HBEGF, RA) and antagonist-driven experiments (TGFB). These experiments collectively link these pathways to MG responses that are dependent on microglia, although the experimentation does not directly resolve the nature of the endogenous signaling dynamics that unfold between MG and microglia following injury.

Comments for the author

"Formation of Müller glia derived progenitors in retinas depleted of microglia"

El-Hodiri et. al. probe regulatory mechanisms of pathways linked to Müller glia (MG) activation following injury that are contingent on microglia presence. This is an incompletely resolved question of high importance to the field of retinal regeneration, as microglia have been documented to exert species-specific and context-specific influences on the outcome of MG reprogramming. A greater understanding of these pathways would prove meaningful to efforts to expand the regenerative competence of mammalian MG.

The authors rely on the chick model of NMDA-induced injury, for which they have characterized microglia-MG interactions to some extent in previous publications. The interrogated pathways largely stem from candidate regulators identified across numerous scRNA-seq datasets. The work presented is significant for the field of retina regeneration as the authors detail a function (or lack thereof) for many of the identified pathways in driving MG proliferation and/or migration in the presence/absence of microglia. These include a series of agonist-driven experiments (WNT, FGF, HBEGF, RA) and antagonist-driven experiments (TGFB). These experiments collectively link these pathways to MG responses that are dependent on microglia, although the experimentation does not directly resolve the nature of the endogenous signaling dynamics that unfold between MG and microglia following injury.

1. There are, however, issues on the presentation of the data that makes it difficult to follow and the interpretation of the data is also not so clear (see comments below) with areas of controversy in several parts when dealing with MG proliferation vs MG nuclei migration In addition, after all the intricacies of the data presented, the reader leaves with not a clear mechanistic view of the microglia-MG regulation during retinal damage. While the authors suggest some of the dynamics that may be at play using *in silico* ligand-receptor prediction tools, these methods are suggestive/tentative in nature, and validation or at least a more in-depth presentation of the underlying gene expression data could help to solidify the observations.

There are minor and major issues that need to be addressed before this work is published in Development.

General

- The title could be more fitting to the data presented- it is too general
- Line 30 Not all authors have a contribution
- LFC, p-values, and/or expression levels of shown genes from scRNA-seq dataset should be provided in figures or provided as supplement, as the underlying values cannot be readily appreciated from row-scaled dot plots.
- For dot plots throughout the manuscript, it is stated that significant genes are included. However, in the absence of these values, it cannot be ascertained between which conditions the change(s) are occurring.
- For all single-cell feature plots, authors should state in the methods or figure what values

© 2023. Published by The Company of Biologists under the terms of the Creative Commons Attribution License (https://creativecommons.org/licenses/by/4.0/).

are shown and what normalization metric was applied (raw counts, log-normalized counts, etc.).

- The effects of the tested compounds on cell death are not assessed, but could be important.
- There are inconsistencies on the summary of each section. In some there is a concluding remark and in others it is lacking (line 430; 467) and in some cases it is just very generic (lines 384-6).
- A summary figure/schematic of the pathways tested and major conclusions would be helpful to orient readers.

Materials and methods

- Software version should be added wherever possible.
    - Authors should consider deposition of raw data into a sequence repository wherever possible, per journal guidelines: "Publication in Development requires that primary data for high-throughput experiments such as microarrays, RNA-seq, ChIP-chip or ChIP-seq be deposited in the appropriate public database. The Gene Expression Omnibus (GEO), ArrayExpress, European Nucleotide Archive (ENA) or Short Read Archive (SRA) are appropriate repositories for most functional genomics data."
- For statistical analysis where parametric tests are employed, authors should state/demonstrate whether appropriate assumptions were met.
- Methods do not contain information on the injection of several compounds mentioned in the abstract. Several fatty acid synthase inhibitors appear to be listed, which are not used in the present study.
- Methods do not contain information on microglia depletion approach.
- A thorough explanation of Seurat workflow should be added to methodology. Specify if integration methods were used across datasets.
- Methods should state how p-value was obtained from ShinyGO and whether multiple hypothesis correction was performed.
- Clarification on how injections of test compounds on the R eye vs vehicle on L eye could affect interpretation of results as there is communication within the optic tectum that could affect the results

Microglia depletion scRNA-seq

- Line 230 as stated should be 82 + 5 = 87
- Figure 1 UMAPs: it is unclear whether microglia were captured in the assay in saline-treated retinas, and whether these cells were absent from clodronate-treated retinas.
- Line 278-279 incomplete sentence.

Ligand-receptor interactions

- Fig. 2a-f as written (Lines 273-280) implies that differences in autocrine signaling networks were identified and attributable to the presence of microglia, based on the detection of significant connections in one condition and failure to detect the same significant connection in another condition. Because the comparison for each condition appears to have been done independently and then subsequently overlapped, it is unclear if this comparison would logically lead to true changes in signaling events. It would be helpful to plot the expression (e.g., violin plots) of key effectors, such as those highlighted in text or Venn diagrams, to show how the expression of these effectors changes between groups and whether changes are actually significant.
- If microglia were also captured in the dataset generated in Fig. 1, it would be logical to identify ligand-receptor pairs between MG and microglia (as opposed to only autocrine signaling), and to see whether these effectors are perturbed in the absence of microglia.
- In general, a supplementary file of the complete ligand-receptor interactions (or table format) could help organize the reader as to which conditions are predicted to exhibit which ligand-receptor connectivity. As is, it is difficult to identify specific pathways and search for presence / absence and the strength of the predicted network across conditions.
- Similar to above comment, it would be helpful to visualize the expression changes of key effectors identified in the Fig. 2G NMDA time course, as well as how they relate to the

© 2023. Published by The Company of Biologists under the terms of the Creative Commons Attribution License (https://creativecommons.org/licenses/by/4.0/).

novel datasets, so that readers can appreciate how these interactions may be perturbed following microglia ablation.
- The last sentence on this section seems to just add extra information but not integrated with the data presented.

WNT regulation in the retina
- Line 295-296 WNT4 & WNT6 add a reference to figure
- Paragraph beginning on line 304 introduces details of a dataset that appears to have been used earlier in the manuscript (in paragraph line 281). This sc dataset was used extensively previously but they do not actually introduce what it is until later in the manuscript.
- Line 352 “REF “?
- Line 353 state the cocktail used in the current manuscript... is this ABC? Define abbreviation.
- Authors demonstrate a very tight up-regulation of WNT4 / WNT6 in response to NMDA within 12 hours (Fig. 3F) to 24 hours (Fig. 2H) following injury, which fails to be realized in the absence of microglia. However, the use of a WNT agonist did not stimulate MGPC proliferation, but instead induced delamination. The authors previously found cell migration to be highly downregulated in NMDA-treated, microglia-depleted retinas. The authors need to consider follow up experimentation to pinpoint a more precise role for the function of endogenous WNT ligands. In addition, it seemed that the number of MG nuclei migrating was much higher than the number of sox-2 + cells present in the non-microglia depleted eyes. Because sox-2 is also a neural retina progenitor marker, it is important to use another marker for MG that could distinguish MG from retinal progenitors if possible (Fig 3K).

FGF regulation in the retina
- Lines 362-369 refers to Fig. 1a but this should likely be Fig. 4a.
- Authors should comment on the apparent rescue of delamination in this section, similar to as was done for other sections.

HBEGF regulation in the retina
- It is unclear how to reconcile the finding line 393 “EGFR was not expressed by MG” with the ligand-receptor networks in Fig. 2 showing predicted EGFR signaling interactions.
- Line 423 “ERBB2 was not widely expressed by different types of retinal cells (Fig. 5e)” does not appear in agreement with the shown feature plot.

SMAD3
- It appears that slight up-regulation of TGFB1/2 are observed in the injured retina in the absence of microglia, although possibly much of the effect could be mediated through inhibitors such as INHBA/ TGIF1. Functional experimentation could be used to more precisely resolve the mechanisms linking these factors to MG proliferation.
- INHBA is listed as “IHBA” in dot plots; authors should verify that this is the correct gene.

RAR
- Figures 7E-F are not discussed in the manuscript.
- For example, there is no discussion of why manipulation RAR signaling does not provoke nuclei translocation- whereas it was stated in the manuscript (lines 548-9) that when there is MG proliferation, there is always migration and it is not the case here.

First revision

Author response to reviewers' comments

According to the comments of the reviewers, the manuscript has been revised as follows. Changes to the manuscript are highlighted in yellow.

Reviewer 1

© 2023. Published by The Company of Biologists under the terms of the Creative Commons Attribution License (https://creativecommons.org/licenses/by/4.0/).

1. Results: Lines 225-246 have a lot of redundancy and should be rewritten for conciseness and clarity.
We have edited the text to reduce redundancy and improve clarity.

2. Relying on clodronate liposomes as the only method to remove microglia is worrisome as it stimulates death of phagocytic cells not just microglia. There is a report indicating that removal of retinal microglia enhances the phagocytic activity of Muller glia (Glia 70:1402, 2022). Additional approaches to ablating retinal microglia are important to convincingly demonstrate the results obtained are due to microglia ablation and not from off-target effects on MG or other phagocytic cell types. Most studies employ PLX3395 or some more recent version of this drug that blocks the csf1r receptor and it would seem this should be applied to this study to ensure off-target effects of clodronate don’t underlie some of their results.
We have applied PLX5622 and BLZ945 to the chick with the intent of ablating microglia and macrophage in the retina. Although chick microglia/macrophage selectively express CSF1R, the above-mentioned CSF1R antagonists did not significantly deplete microglia/macrophage within the chick retina. It is possible that the survival of chick microglia does not depend on activation of CSF1R or the specificity of the antagonists to chick CSF1R is low.
We have previously demonstrated the time-course and specificity of fluorescently-labeled clodronate liposomes, wherein these liposomes accumulate only at the vitread surface of the retina and are taken-up by microglia, and the MG appear entirely unaffected by the clod-liposomes (Zelinka et al., 2012). Although there is some evidence that MG in the fish retina can adopt some phagocytic function (Thiel et al., 2022), to the best of our knowledge, there is no evidence to suggest that MG in bird or mammalian retinas have phagocytic functions. Accordingly, we probed our scRNA-seq libraries for receptors associated with the phagocytic cup and early endosomes in MG in NMDA-damaged retinas with and without microglia. MG did not upregulate any receptors found in phagocytic cups or genes associated with early endosomes in damaged retinas where microglia have been ablated. These genes are either not expressed (or expressed at very low levels) or unaffected by the ablation of microglia in retinas, with the exception of BIN1 and RABEP1 (only 2 out of 15 genes; AP4M1 was not significantly different). These findings suggest that MG do not adopt phagocytic function in damaged chick retinas. We have added text to pages10 and 11 to better describe the specificity of the clodronate liposome in the eyes of chicks and evidence suggesting that MG in chick retina do not acquire phagocytic functions in the absence of microglia.

3. Fig. 2 suggests tgfb-smad3 signaling takes place between microglia and MG in the injured retina; however, in the chlodrinate treated retinas that lack microglia, they employ Tgfb/Smad inhibitor (SIS3) to see if they can rescue the proliferation defect resulting from microglia ablation, but these chlodrinate treated retinas don’t have microglia. This experiment does not make sense.
Normally microglia produce TGFB1 that may activate signaling in MG through SMAD3. These data are illustrated in Figure 2g and schematic summary Fig. 8. However, we present data in Figure 6b that MG significantly and highly upregulate TGFB2/3 in damaged retinas, maintain high levels of TGFB2/3 when microglia are ablated, and downregulate inhibitors TGIF1 and INHBA in damaged retinas missing microglia. These data suggest that there is increased MG autocrine signaling involving TGFb-SMAD3 in damaged retinas missing microglia, thereby providing rational for inhibiting TGF/SMAD3. We have added text to page 21 to better describe the rationale for these experiments.

4. What receptors mediate the action of Fgf2 and Hbegf in the chick retina? Please show ligand and receptor gene expression from the scRNAseq data so one can determine the cell types and level of expression of putative ligand receptor pairs. Since they both act to stimulate MG progenitor formation in absence of microglia, do they converge on the same signaling cascade? It seems the data is suggesting these ligands activate receptors on MG that are normally engaged by factors released from microglia. What microglia candidates fit these criteria and what happens when they are overexpressed or introduced (recombinant protein) in the chick retina? Also, why are Fgf ligands downregulated when it seems the authors are proposing Fgf signaling is critical to MG proliferation. What is the endogenous ligand if Fgf2 is not normally expressed in the chick retina?
In Figures 4a,e,f evidence is that FGF1, FGF10 and FGF12 are widely expressed by cells in the chick retina, and levels of FGFR1, MAPK6 and MAPKAPK2 are significantly decreased in MG in damaged retinas where microglia have been ablated. We have added text to page 18 to better describe these data.

© 2023. Published by The Company of Biologists under the terms of the Creative Commons Attribution License (https://creativecommons.org/licenses/by/4.0/).

5. Does apoptosis correlate with the different treatments affecting MG proliferation in injured and microglia-depleted retinas?
The effects of tested compounds and microglia ablated upon cell death have been thoroughly studied previously. The ablation of microglia from NMDA-damaged chick retinas suppresses, and delays, numbers of dying neurons (Fischer et al., 2015 Glia). FGF2 is potently neuroprotective in the chick retina (Fischer, Scott 2009 Glia). HBEGF has no effect on numbers of dying cells in retinas treated with NMDA damaged retinas (Todd et al., 2015). The cocktail of GSK3b-inhibitors has no effect upon numbers of dying cells in damaged retinas (Gallina et al., 2016). Interestingly, exogenous TGFB2 increased levels of cell death whereas SIS3 has no effect on cell death (Todd et al., 2017 Glia). We found that treatment of damaged retinas with drugs that target RAR receptors (TTNBP or BMS493) or drugs elevate retinal levels of RA by inhibiting degradation (liarozole), had no effect upon cell death in damaged retinas (Todd et al., 2018). In short, growth factors or small molecules inhibitors have been shown to either have no effect or reduce numbers of dying cells and diminish retinal damage, which is expected to potentially reduce numbers of MGPCs. However, these growth factors and small molecule inhibitors all increased numbers of proliferating MGPCs, with the exception of the cocktail of GSK3b inhibitors. We have added text to the Discussion page 28 to describe these findings.

6. Wnt4 and 6 are the most highly down-regulated genes when microglia are ablated. Does this correlate with changes in MG nuclear b-catenin?
7. Does microglia ablation result in changes in b-catenin nuclear localization in the injured retina? Also, it would be nice to show the impact Gsk3b inhibition has on b-catenin nuclear localization.
These are very good questions that we have tried to investigate. We applied multiple different lots of DSHB supernatant and ascites fluid of anti-nuclear Beta-catenin and none of these lots have produced labeling. This has been frustrating since this monoclonal has worked for us in the past (Gallina et al., 2016). In this previous report, we demonstrated that GSK3b inhibitors potently stimulate the accumulation of nuclear B-catenin in MG in damaged retinas (Gallina et al., 2016). These are good questions that we have, unfortunately, been unable to investigate in the current study.

8. Previous studies suggested a role for NFkb signaling in mediating the effects of microglia and MG. How does Fgf2 and Hbegf affect Nfkb signaling in MG? Are they acting upstream or downstream of Nfkb signaling? What happens if add Nfkb inhibitors and treat retinas with either Fgf2 or Hbegf in microglia-depleted, injured retinas?
In chick retinas where microglia have been ablated, NFkB antagonists are unable to rescue MGPC proliferation, whereas NFkB antagonists stimulate MGPC proliferation in damaged retinas with microglia present. Interestingly, NFkB agonists (or TNF-related ligands) that suppress MGPC proliferation in damaged retinas with microglia, potently rescue MGPC proliferation in microglia-depleted chick retinas (Palazzo et al., 2020). These findings have suggested that a brief, rapid activation of NFkB in MG is required to activate the process of reprogramming into MGPCs (Palazzo et al., 2020, 2022), including activation or enabling of FGF/HGEGF/MAPK signaling. We have added text to better describe these findings and a schematic summary in Fig. 8.
We have applied at least 10 different phospho-specific antibodies to different NFkB pathway components, and none of these antibodies have produced plausible labeling in chick or mouse retinas. Thus, we are unable to determine whether NFkB signaling is downstream of FGF2 or HBEGF in the chick retina. In the mouse retina, FGF2 has no significant activation of NFkB reporter in the mouse retina (Palazzo et al., 2023). Further, MG-specific conditional knock-out of Ikkb causes a significant downregulation of MAPK-signaling gene modules in damaged mouse retinas, suggesting that activation of MAPK gene networks are downstream of NFkB in activated MG (Palazzo et al., 2022). We have added text to page 25 to indicate that upregulation of MAPK gene networks appears to be downstream of NFkB, and FGF2/MAPK do not activate NFkB signaling in MG.

9. The title is misleading and implies MG-derived progenitors are formed independent of microglia; rather, the paper indicates microglia are necessary for MG progenitor formation and that if microglia are depleted this can be rescued by Fgf2 or Hbegf treatments. Maybe something like: Fgf2 and Hbegf rescue MG progenitor formation in the microglia-depleted chick retina.
We have edited the title to better describe the main findings of the paper.

© 2023. Published by The Company of Biologists under the terms of the Creative Commons Attribution License (https://creativecommons.org/licenses/by/4.0/).

Minor comments
All minor comments have been corrected

10. Why are there no microglia in the UMAP plots for Fig. 1a, b?
This UMAP object in Figure 1 was generated using 10X Chromium 3' V2 reagents (with reduced sensitivity compared to newer reagents). Accordingly, following filtering for doublets and cells with few genes per cells, there were no microglia from control retinas. This is consistent with first publications using scRNA-seq libraries or chick retinas (Hoang et al., 2020; Palazzo et al., 2020; Campbell et al., 2019) wherein microglia were not captured/annotated within Seurat-filtered objects. We have added text to page 11 to describe the capture of microglia in these scRNA-libraries.

12. Fig. 2e, f: what is the significance of LR pairs shown next to the pie charts?
Representative LR-interactions unique to MG in undamaged retinas with and without microglia (e) and NMDA damaged retinas with and without microglia (f). We have modified text in the Results and revised figure 2 to better describe the LR pairs.

14. In the discussion (line 549) they say proliferation is always associated with nuclear migration, but both with Smad3 inhibition and RARa activation, they see a small but significant increase in proliferation which is not coupled with delamination.
In the current study we observed modest increases in MGPC proliferation in damaged retinas missing microglia treated with RAR agonist or Smad3 inhibitor, and this modest increase in proliferation was associated with modest delamination of MG nuclei. We have added text to the Results to better describe the correlation between levels of MGPC proliferation and levels of nuclear delamination.

Reviewer 2
1.Better clarity around the use of the term microglia versus microglia/macrophages is needed. This is not clarified until the beginning of the results where it is stated that the term "microglia" will refer to both. It should be more clear in the title, abstract and introduction that both populations are potentially depleted/involved. Also, in the methods it is not clear if the single cell markers listed for microglia are also interpreted as applying to macrophages. Given the growing appreciation for the distinction between these cell populations it is important to be accurate in how they are referred to. It is pretty squishy throughout the paper which is problematic.
We have better defined our word usage regarding microglia and macrophage in the Abstract and Introduction. We have added text to the Methods to indicate that markers that we used to identify microglia do not distinguish between microglia and macrophage. In our experience in the chick retina, we have not identified markers that unambiguously distinguish between microglia and infiltrating macrophage. Markers, such as high levels of CD45 or CCR2, which have been used to distinguish macrophage in the mammalian retina do not distinguish macrophage in the chick retina.

2.In the abstract, a bit more context in a few spots would help the reader. For example, since the study is in chick, after the first sentence it would be helpful to state something like: "In chick, depletion of microglia prevents formation of proliferating MGPCs in response to damage." Also, stating at the outset that the analysis focused on changes in signaling pathways would give more focus to the results and conclusions.
The abstract has been rewritten to better describe the context of the chick retina and cell signaling pathways.

3.Figure 1 – are microglia/macrophages identified in the scRNAseq data? One would expect that they would be present in the saline samples. They are not identified in the figure and not mentioned. Is there evidence of depletion from the data? As noted above, markers for microglia are mentioned in the methods section describing analysis of single cell data. Since depletion precedes damage, is there evidence for infiltration of macrophages in response to damage?
The scRNA-seq libraries illustrated in Figure 1 were generated using Chromium 3' V2 reagents and very few (<50) microglia were captured from control (saline and no clodronate liposomes). Accordingly, to investigate the transcriptomic profiles of microglia we analyzed scRNA-seq databases generated using newer, more sensitive reagents to yield more than 1100 microglia. We have reported previously that infiltration of macrophage and the repopulation of microglia in damaged retinas treated with clodronate-liposome begins to occur in the far periphery and near the

© 2023. Published by The Company of Biologists under the terms of the Creative Commons Attribution License (https://creativecommons.org/licenses/by/4.0/).

pecten at about 4 weeks after treatment (Zelinka et al., 2012; Fischer et al., 2014). Further, evidence is provided that damaged microglia-depleted retinas are completely devoid of CD45+ monocytes for the duration of experimentation (Fig. 4b). We have added text to pages 10 and 17 to address these concerns.

4.Line 269-271 is confusing: “Numbers of LR- interactions (significant upregulation of putative ligand and receptor) between cell types in the different treatment groups varied between 70 and 315.” Looking at the figure it is not clear where these numbers come from.
We have revised this text to describe the number of autocrine LR-interaction among MG from different treatment groups which includes between 140 to 211 LR-interactions, and we have included the full lists of LR-interactions as supplemental data (table 6) (as requested by Reviewer #3).

5.Line 276: Fig. 2a,b,f should instead read: Fig. 2a,b,e. In general, the description of Figure 2a-f (lines 273-280) is confusing and should be clarified and tightened up. For example, this is not a sentence: “When microglia are ablated (Fig. 2a, e).” And the description jumps from resting to activated then apparently back to resting MG again. It is difficult to follow the signaling pathways being described on the chord diagrams, and the description didn’t always seem to correspond to the figure. This section needs improvement, and the differences between the conditions in the chord diagrams could be indicated in some way.
We have extensively revised this section to improve clarity.

6.Figure 2g described analysis of scRNAseq data at 3, 12 and 48 hrs after damage. Was this experiment done for this study? It is not described anywhere in the methods and data are not shown in the results before mention in this panel.
We have added text to better describe the previous use of these libraries, which are used in many figures in the current study.

The legend for Figure 2g states: “Representative significant LR-interactions between microglia and MG are illustrated for undamaged and damaged retinas with or without microglia.” This seems inaccurate. There can be no interactions between microglia and MG without microglia.
This error has been corrected to “Representative LR-interactions between microglia and MG are illustrated for undamaged and damaged retinas at different times after NMDA-treatment (g).” These data were anaylzed for scRNA-libraries were microglia well-represented.

7.Lines 295-296: “Wnt-ligands, WNT4 and WNT6, are among the most significantly downregulated genes in MG in damaged retinas where microglia were ablated.” Are there data shown to support this statement? No figure, panel or table are referred to. This seems to be the rationale for the pivot to the analysis of Wnt pathways in Figure 3.
We added reference to Figure 1g,h and supplemental table 2 to better indicate the rational for Figure 3.

8.Lines 324-325 state: “…we probed large aggregate scRNA-seq library of MG (>70,000 cells) with glia bioinformatically isolated from retinas….” The source of these data are entirely unclear. Does this represent yet another scRNA-seq experiment? With enough sequencing to yield 70,000 MG for analysis? That is how this reads. None of this is described in the results or methods. If this is from previously published data there is no citation or GEO dataset identified.
To gain a more comprehensive understanding of patterns of expression of Wnt-related genes, we probed a large aggregate scRNA-seq library of MG (>70,000 cells) generated as described in previous studies (Hoang et al., 2020; Campbell et al., 2021a; Campbell et al., 2021b; El-Hodiri et al., 2022). This scRNA-seq dataset was deposited in GEO (GSE135406).

9.The title may not best capture the focus of this study. The analysis alternates between effects on MG of microglia/macrophage depletion, and also effects on retinal gene expression and MG at different times after damage (eg. Figures 3-6). This actually makes it difficult to read since the rationale for the transitions are not always well explained. The more unifying theme is the focus on analysis of signaling pathways under these conditions.
We have revised the title to better describe the main findings of the paper. We have edited many portions of the manuscript to improve clarity and readability.

© 2023. Published by The Company of Biologists under the terms of the Creative Commons Attribution License (https://creativecommons.org/licenses/by/4.0/).

10.Lines 435-436: “We probed for changes in expression levels of TGFB-related genes in MG in normal and damaged retinas, with and without retinas”. Correction needed.
This has been corrected to “with and without microglia.”

11.Primary single cell RNA-seq data files should be submitted to NCBI GEO.
Sequences have been uploaded to GEO (GSE135406, GSE242796)

Reviewer 3
• The title could be more fitting to the data presented- it is too general
The title has been modified to better describe the main point of the paper.

• Line 30 Not all authors have a contribution
This error has been corrected

• LFC, p-values, and/or expression levels of shown genes from scRNA-seq dataset should be provided in figures or provided as supplement, as the underlying values cannot be readily appreciated from row-scaled dot plots.
We have added supplemental tables to include the adjusted p-values for the dotplots. These data were included in Supplemental tables 1,2,3 and 5 for all dotplots for MG from undamaged retinas ± microglia and damaged retinas ± microglia.

• For dot plots throughout the manuscript, it is stated that significant genes are included. However, in the absence of these values, it cannot be ascertained between which conditions the change(s) are occurring.
We have added supplemental tables with detailed statistical analyses between different treatment groups.

• For all single-cell feature plots, authors should state in the methods or figure what values are shown and what normalization metric was applied (raw counts, log-normalized counts, etc.).
We have included additional details for Cell Ranger and Seurat workflows to better detail the bioinformatics underlying the different analyses.

• The effects of the tested compounds on cell death are not assessed, but could be important.
This point was addressed above; reviewer #1 point 5.

• There are inconsistencies on the summary of each section. In some there is a concluding remark and in others it is lacking (line 430; 467) and in some cases it is just very generic (lines 384-6).
We have added text to summarize each section in a consistent manner to include insightful concluding remarks.

• A summary figure/schematic of the pathways tested and major conclusions would be helpful to orient readers.
We have created a new schematic summary (Fig. 8)

Materials and methods
• Software version should be added wherever possible. Authors should consider deposition of raw data into a sequence repository wherever possible, per journal guidelines: "Publication in Development requires that primary data for high-throughput experiments such as microarrays, RNA-seq, ChIP-chip or ChIP-seq be deposited in the appropriate public database. The Gene Expression Omnibus (GEO), ArrayExpress, European Nucleotide Archive (ENA) or Short Read Archive (SRA) are appropriate repositories for most functional genomics data."
We have revised the Methods to include additional details of bioinformatic analyses including software versions. Sequences have been uploaded to GEO (GSE135406, GSE242796)

• For statistical analysis where parametric tests are employed, authors should state/demonstrate whether appropriate assumptions were met.
We have revised to statistical analyses to include a Levene’s test was to determine whether data from control and treatment groups had equal variance. For treatment groups where the Levene’s test indicated unequal variance, a Mann-Whitney U test was used.

© 2023. Published by The Company of Biologists under the terms of the Creative Commons Attribution License (https://creativecommons.org/licenses/by/4.0/).

• Methods do not contain information on the injection of several compounds mentioned in the abstract. Several fatty acid synthase inhibitors appear to be listed, which are not used in the present study.
This error had been corrected

• Methods do not contain information on microglia depletion approach.
This error had been corrected

• A thorough explanation of Seurat workflow should be added to methodology. Specify if integration methods were used across datasets.
Text has been added to the Methods to better describe the Seurat workflow and parameters used to generate UMAP clusters of cells.

• Methods should state how p-value was obtained from ShinyGO and whether multiple hypothesis correction was performed.
The adjustment for multiple hypothesis testing is performed through ShinyGo by normalizing the enrichment score for each gene set to account for the size of the set, to produce a normalized enrichment score. The proportion of false positives is controlled by calculating the false discovery rate (FDR) corresponding to each NES. The FDR enrichment p-value is calculated as the estimated probability that a set with a given NES represents a false positive finding; it is computed by comparing the tails of the observed and null distributions for the NES (Ge et al., 2020).

• Clarification on how injections of test compounds on the R eye vs vehicle on L eye could affect interpretation of results as there is communication within the optic tectum that could affect the results
We assume that the reviewer is suggesting the possiblilty of neuronal communication between retinas. Indeed, the chick retina receives a mono-synaptic projections from the contralateral retina. Efferent projections to the retina, from the isthmo-optic nucleus, make one-to-one connections to about 10,000 efferent targets cells per retina, but only in ventral regions of the retina (Fischer et al., 1999; Morgan et a., 1994; Nickla et al., 1994; Paes de Carvalho et al., 1996). In the chick, efferent target cells can be easily identified based on their unique morphology, relatively large size and label for parvalbumin and nNOS (Fischer et al., 1999). The efferent target cells appear to be an atypical type of amacrine cell that is resistant to excitoxicity (Fischer et al., 1999) and provide GABAergic inhibitory input to other types of retinal neurons along the proximal borders of the INL (Lindstrom et al., 2010). It is difficult to imagine how these the efferent target cells significantly impact the microglia or MG in the chick retina. Further, treatment groups received growth factors or small molecule agonists/antagonists which seem unlikely to activate ganglion cells that project to the isthmo-optic nucleus to activate efferent projections and efferent target cells in the contralateral retina and impact the formation of MGPCs.

Microglia depletion scRNA-seq
• Line 230 as stated should be 82 + 5 = 87
This have been corrected

• Figure 1 UMAPs: it is unclear whether microglia were captured in the assay in saline-treated retinas, and whether these cells were absent from clodronate-treated retinas.
Very few (<50) microglia were captured in the control (no clodronate) libraries. No microglia were captured in libraries of clodronate-treated retina, as expected. We have added text to better describe these data.

• Line 278-279 incomplete sentence.
This has been corrected.

Ligand-receptor interactions
• Fig. 2a-f as written (Lines 273-280) implies that differences in autocrine signaling networks were identified and attributable to the presence of microglia, based on the detection of significant connections in one condition and failure to detect the same significant connection in another condition. Because the comparison for each condition appears to have been done independently and then subsequently overlapped, it is unclear if this comparison would logically lead to true changes in signaling events. It would be helpful to plot the expression (e.g., violin plots) of key

© 2023. Published by The Company of Biologists under the terms of the Creative Commons Attribution License (https://creativecommons.org/licenses/by/4.0/).

effectors, such as those highlighted in text or Venn diagrams, to show how the expression of these effectors changes between groups and whether changes are actually significant.
All differentially expressed genes (DEGs), adjusted p-values and percent of expressing cell are included in supplemental tables 1,2 and 3. These DEGs were utilized by SingleCellSignalR to identify putative LR-interactions. The identification of lists of DEGs must be performed independently for each experimental condition and compared for MG in retinas ± damage and ± microglia. We have included the full lists of LR-interactions applied to Venn diagrams as supplemental data (supplemental Table 6).

• If microglia were also captured in the dataset generated in Fig. 1, it would be logical to identify ligand-receptor pairs between MG and microglia (as opposed to only autocrine signaling), and to see whether these effectors are perturbed in the absence of microglia.
Very few (<50) microglia were captured in the control, no clodronate, libraries. However, we analyzed LR-interaction between microglia and MG in a different aggregate library wherein we captured more than 1100 microglia. The LR-interactions are illustrated in Figure 2g. We have added text to better describe these data.

• In general, a supplementary file of the complete ligand-receptor interactions (or table format) could help organize the reader as to which conditions are predicted to exhibit which ligand-receptor connectivity. As is, it is difficult to identify specific pathways and search for presence / absence and the strength of the predicted network across conditions.
We have included the full lists of LR-interactions as supplemental table 6.

• Similar to above comment, it would be helpful to visualize the expression changes of key effectors identified in the Fig. 2G NMDA time course, as well as how they relate to the novel datasets, so that readers can appreciate how these interactions may be perturbed following microglia ablation.
Changes in expression of key effectors are provided in subsequent figures in panels with dot plots. We have added supplemental tables to provide specific (Supplemental table 5) and general (Supplemental table 4) statistics for LogFc change and adjusted p-values.

• The last sentence on this section seems to just add extra information but not integrated with the data presented.
We have added a sentence to sum-up and integrate the data on page 14.

WNT regulation in the retina
• Line 295-296 WNT4 & WNT6 add a reference to figure
We added a reference to Fig. 1g,h; supplemental Tables 2 and 4.

• Paragraph beginning on line 304 introduces details of a dataset that appears to have been used earlier in the manuscript (in paragraph line 281). This sc dataset was used extensively previously but they do not actually introduce what it is until later in the manuscript.
We have added text to page 14 to better describe the origin and previous use of this scRNA-seq dataset.

• Line 352 “REF “?
This has been corrected

• Line 353 state the cocktail used in the current manuscript... is this ABC? Define abbreviation.
We have added a definition of the ABC to the methods.

• Authors demonstrate a very tight up-regulation of WNT4 / WNT6 in response to NMDA within 12 hours (Fig. 3F) to 24 hours (Fig. 2H) following injury, which fails to be realized in the absence of microglia. However, the use of a WNT agonist did not stimulate MGPC proliferation, but instead induced delamination. The authors previously found cell migration to be highly downregulated in NMDA-treated, microglia-depleted retinas. The authors need to consider follow up experimentation to pinpoint a more precise role for the function of endogenous WNT ligands. In addition, it seemed that the number of MG nuclei migrating was much higher than the number of sox-2 + cells present in the non-microglia depleted eyes. Because sox-2 is also a neural retina progenitor marker, it is

© 2023. Published by The Company of Biologists under the terms of the Creative Commons Attribution License (https://creativecommons.org/licenses/by/4.0/).

important to use another marker for MG that could distinguish MG from retinal progenitors if possible (Fig 3K).
We applied recombinant Wnt4 to normal or damaged retinas with and without microglia. This had no effect likely because the recombinant Wnt4 was not biologically active without proper folding and ligations. We are unaware of sources of purified biologically active Wnt4 or Wnt6. We have added text to page 17 to mention these negative data. We applied additional markers, Pax2, Pax6 and Glutamine Synthetase (GS) for MG that are known to change during reprogramming into proliferating MGPCs. In response to GSK3b inhibitor, we found that levels of Pax2 were not significantly affected whereas levels of GS were significantly decreased and levels of Pax6 appeared increased. Collectively, these findings indicate that activated microglia in damaged retinas stimulate MG to highly upregulate Wnt-ligands, and Wnt-signaling may normally stimulate the migration and de-differentiation of MG in damaged retinas, but this is not sufficient to stimulate the proliferation of MGPCs unless other microglia-dependent signals are provided. Supplemental Fig. S3 and text (page 18) have been added to describe these findings and interpretations.

FGF regulation in the retina
• Lines 362-369 refers to Fig. 1a but this should likely be Fig. 4a.
This has been corrected

• Authors should comment on the apparent rescue of delamination in this section, similar to as was done for other sections.
We have added statements to mention whether the delamination of MG/MGPC nuclei was rescued by the different treatments.

HBEGF regulation in the retina
• It is unclear how to reconcile the finding line 393 “EGFR was not expressed by MG” with the ligand-receptor networks in Fig. 2 showing predicted EGFR signaling interactions.
We have corrected this error on page 20 as follows: Although EGFR was expressed at very low levels, there was a significant increase in MG undamaged retinas when microglia were ablated (Fig. 5a,b, supplemental table 1 and 5). The large and highly significant changes in levels of ligand (HBEGF) coupled to very low levels of expression of receptors has driven detection of this LR-interaction in the SingleCellSignalR analyses.

• Line 423 “ERBB2 was not widely expressed by different types of retinal cells (Fig. 5e)” does not appear in agreement with the shown feature plot.
We have corrected this error.

SMAD3
• It appears that slight up-regulation of TGFB1/2 are observed in the injured retina in the absence of microglia, although possibly much of the effect could be mediated through inhibitors such as INHBA/ TGIF1. Functional experimentation could be used to more precisely resolve the mechanisms linking these factors to MG proliferation.
The upregulation of TGFB1/2 is not significant, whereas downregulation of INHBA and TGIF1 were significantly downregulated. Indeed, it is likely that increased signaling through TGFB/Smad is mediated by decreases in inhibitors. We have added text to page 22 to better describe the rational for these experiments.

• INHBA is listed as “IHBA” in dot plots; authors should verify that this is the correct gene.
This error has been corrected in Figures 3b and 3f.

RAR
• Figures 7E-F are not discussed in the manuscript.
We have added text the Results to describe the data illustrated in figures 7e,f.

• For example, there is no discussion of why manipulation RAR signaling does not provoke nuclei translocation- whereas it was stated in the manuscript (lines 548-9) that when there is MG proliferation, there is always migration and it is not the case here.
We have added text to the Results (page 23) to better describe how nuclei translocation is usually (with the interesting exception of GSK3B inhibition) correlated with levels of proliferation. Since

© 2023. Published by The Company of Biologists under the terms of the Creative Commons Attribution License (https://creativecommons.org/licenses/by/4.0/).

the treatment with the RAR agonist had a very modest effect on proliferation, there was a modest effect upon nuclear translocation.

Second decision letter

MS ID#: DEVELOP/2023/202070

MS TITLE: Formation of Muller glia-derived progenitor cells in retinas depleted of microglia

AUTHORS: Heithem El-Hodiri, James R Bentley, Alana G Reske, Olivia B Taylor, Isabella Palazzo, Warren A Campbell, Nicklaus R Halloy, and Andy J Fischer

I have now received the reports of two of the referees who reviewed the earlier version of your manuscript and I have reached a decision. The referees' comments are appended below, or you can access them online: please go to BenchPress and click on the 'Manuscripts with Decisions' queue in the Author Area.

The reviewers' overall evaluation is very positive and we would like to publish the revised manuscript in Development. There is a remaining minor query from referee 3 regarding the use of two versions of Seurat. Could you attend to this comment in the manuscript and provide a response in an accompanying letter.

Reviewer 2

Advance summary and potential significance to field

This study by El-Hodiri and colleagues provides a potentially valuable addition to our understanding of how microglia/macrophages contribute to formation of Muller glia-derived progenitor cells in damaged chick retina by use of scRNAseq to analyze gene expression changes in Muller glia after depletion of microglia/macrophages both with and without damage. The focus on analysis of signaling pathways is relevant and key signals are tested for their ability to rescue the effects of microglia/macrophage depletion.

Comments for the author

The authors have thoroughly addressed the suggestions and comments from the reviews. The changes are clearly marked making the changes easy to evaluate. Additional rigor and attention to experimental detail, as well as clarification in key sections of the manuscript have significantly improved the paper. I have no further concerns. The work is a nice addition to the field.

Reviewer 3

Advance summary and potential significance to field

The work presented is significant for the field of retina regeneration as the authors detail a function (or lack thereof) for many of the identified pathways in driving MG proliferation and/or migration in the presence/absence of microglia.
These include a series of agonist-driven experiments (WNT, FGF, HBEGF, RA) and antagonist-driven experiments (TGFB). These experiments collectively link these pathways to MG responses that are dependent on microglia.

Comments for the author

All revisions are to the satisfaction of the reviewer.
Please note:
Seurat version 3 was used for some datasets, where as version 7 was used in others. Default parameters have changed between these versions, such as the inclusion of intronic reads in counts

© 2023. Published by The Company of Biologists under the terms of the Creative Commons Attribution License (https://creativecommons.org/licenses/by/4.0/).

for version 7 and later. The authors should state any arguments used clearly to promote reproducibility of findings.

Second revision

Author response to reviewers' comments

Reviewer #3 asks us to indicate the version of Seurat (V3 or V7) used with respect to annotating and counting genes, and calling cells. However, 10X Cell Ranger is used to annotate and count genes. We added details to the Methods to indicate that 10X Cell Ranger V7 was used to generate the data in Fig.2, whereas 10X Cell Ranger V3 was used to generate the data in the remaining figures.

Third decision letter

MS ID#: DEVELOP/2023/202070

MS TITLE: Formation of Muller glia-derived progenitor cells in retinas depleted of microglia

AUTHORS: Heithem El-Hodiri, James R Bentley, Alana G Reske, Olivia B Taylor, Isabella Palazzo, Warren A Campbell, Nicklaus R Halloy, and Andy J Fischer
ARTICLE TYPE: Research Article

I am delighted to tell you that your manuscript has been accepted for publication in Development, pending our standard ethics checks.

© 2023. Published by The Company of Biologists under the terms of the Creative Commons Attribution License (https://creativecommons.org/licenses/by/4.0/).
